# Exploring developmental assignments shaping experience-driven acquisition of leadership competencies in young clinicians

**DOI:** 10.1186/s12909-022-03544-y

**Published:** 2022-06-28

**Authors:** Mashaal Sabqat, Rehan Ahmed Khan, Raheela Yasmin, Usman Mahboob

**Affiliations:** 1 Department of Health Professions Education, National University of Medical Sciences, Block C Police Foundation, Rawalpindi, Punjab, 968 Pakistan; 2 Department of Surgery, Riphah International University, Rawalpindi, Pakistan; 3 Medical Education and Dean Riphah Academy of Research and Education [RARE], Department of Medical Education, Riphah International University, Rawalpindi, Pakistan; 4 Department of Medical Education, Institute of Health Professions Education & Research, Khyber Medical University, Peshawar, Pakistan

**Keywords:** Experience-driven leadership development, Developmental assignments, Qualitative research, Young clinicians, Medical education

## Abstract

**Background:**

Experiential leadership development is well documented in the corporate sector, but those models cannot be applied as is, in the healthcare domain. The current study proposes a framework for the healthcare sector to enable experiential leadership development for young clinicians. The authors identify developmental assignments (DAs) and explore those characteristics [developmental assignment characteristics; DACs] therein that help develop leadership competencies in young clinicians.

**Methods:**

As part of a qualitative exploratory study in Pakistan, the authors conducted 16 semi-structured interviews with senior clinicians in leadership positions with post-graduate residents under their supervision from different medical specialties. The participants were selected through purposive sampling, ensuring a maximum variation sample. Focusing on participants’ experiences and perspectives related to experience-driven leadership development, the authors used a multi-level theoretical framework for analysis.

**Results:**

The thematic analysis resulted in 19 subthemes with four overarching themes for both objectives. The authors categorized the developmental assignments (DAs) into clinical, academic, and administrative assignments. These assignments can be utilized for leadership development by ensuring that they have the requisite characteristics built into their context and structure. These developmental assignment characteristics (DACs) can range from learner-driven to supervisor-driven. The learner-driven characteristics include autonomy, high levels of responsibilities, unfamiliar assignments, working across boundaries, managing diversity, making a commitment, and creating change. The supervisor-driven characteristics include briefing, debriefing, accountability, and learner-assignment matching. The authors also developed a learner-assignment matching (LAM) framework to guide supervisors in customizing and adjusting the level of each DAC in a DA.

**Conclusion:**

A modern healthcare educational system can utilize studies like this to enable supervisors to develop required leadership skills in young clinicians along with clinical skills.

**Supplementary Information:**

The online version contains supplementary material available at 10.1186/s12909-022-03544-y.

## Introduction

**Fig. 1:**
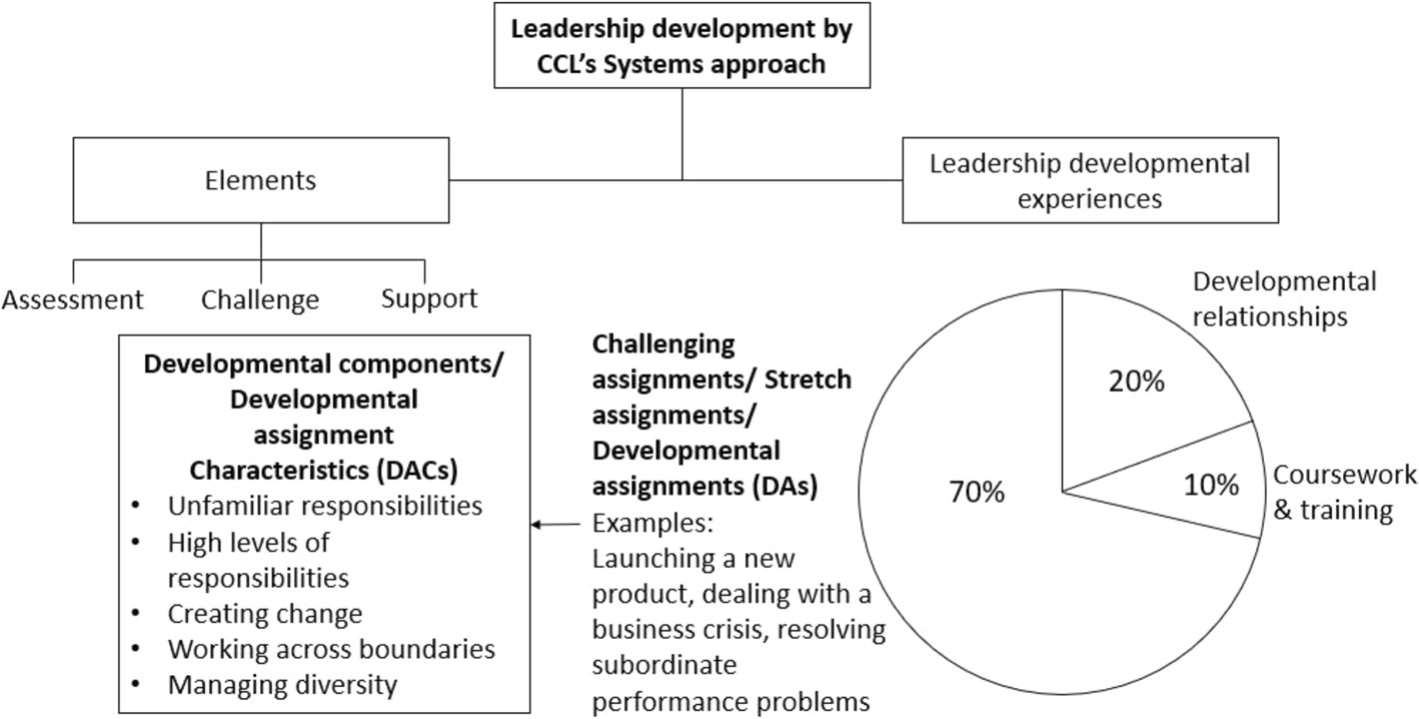
Conceptual framework of the study

Effective modern healthcare systems require professional clinicians to be equipped with leadership skills [1], on top of clinical skills [2], even when they are not in formal management positions. The importance of such leadership skills in the healthcare sector is evident from several recent studies [3, 4]. Thus, many leadership development programs for young clinicians exist in healthcare education. These programs primarily employ classroom-based learning as an educational intervention, with some also using formal work-based approaches such as action learning, coaching, mentoring [5]. While the higher impact of these experiential programs, compared to classroom-based learning, in developing leadership skills is well established in research [6], they come with a challenge that can limit their feasibility in the healthcare setting. The challenge is that young clinicians and their supervisors already have very packed schedules, which leaves less room for specialized tasks to be created and assigned [7, 8]. The current study explores a solution to this issue by proposing an experiential learning system which does not require specialized tasks to be used for leadership development and instead utilizes existing job assignments for this. This solution is based on a series of studies by Morgan McCall and his colleagues [9], at the Center for Creative Leadership [CCL] who propose a model based on the Systems approach to leadership development (Fig. 1). Their work which shows that by adding certain characteristics like assessment, challenge, and support, to regular job assignments, these can be turned into experiential leadership development assignments that impart leadership skills. McCall’s study shows that leadership development occurs within three clusters of experience. The highest impact comes from challenging assignments (70%), followed by developmental relationships (20%) and coursework and training (10%). Researchers like Tracy Duberman [10], and Judy McKimm [11], have recommended the application of McCall’s model for developing leadership skills in young clinicians. However, unlike the corporate sector, identifying and exploring which challenging assignments can be utilized for this remains an under-researched area in medical education. In contrast, this is a well-researched area in the corporate sector, where Ohlott [12], explores using Challenging/ Stretch/ Developmental Assignments (DAs) and defines them as:

“*On-the-job tasks that lead to leadership development in young clinicians. These tasks present a challenge or complexity that stretch the learner and enhance his/her leadership capabilities*”.

Ohlott further elaborates this by presenting five characteristics that when added to a routine job assignment, convert it into a developmental assignment [DA] that develops leadership capabilities.

However Ohlott’s DACs cannot be directly used in the healthcare sector due to the inherent difference the healthcare sector has with the corporate sector [13]. An example of this is the significantly higher cost of mistakes in the healthcare sector with graver consequences in terms of patient safety, compared to the corporate sector.

This is the gap that the current study has aimed to resolve by identifying DACs and exploring how they can be added to young clinicians’ job assignments to convert them into DAs, to develop a framework that can be applied in a healthcare setting considering the specific requirements and constraints applicable there. The findings of this study can enable senior clinicians to develop leadership skills in young clinicians without further burdening the latter’s already busy schedules, by simply restructuring their existing job assignments to include relevant DACs, suitable for the healthcare setting, as described above.

## Methods

### Confidentiality and ethical considerations

Ethical approval was obtained by the ethics review committee, Islamic International Medical College, Islamabad, Pakistan for the period spanning February, 2019 to February, 2020. Written informed consent was obtained from all participants, and participation was kept voluntary. The names of the participants were coded for confidentiality.

### Study design

Given that experiential leadership development in healthcare is considered to be an under-researched area [14], the authors designed a qualitative exploratory study following constructivist research paradigm [15].

### Setting

The study was conducted among senior clinicians in leadership positions in different cities of the Punjab province in Pakistan in both public and private sector healthcare organizations. The authors meant to ensure that the sample would comprise experienced medical experts who had post-graduate residents under their supervision.

Post graduate medical education is based on supervised apprenticeship in these setups with learners relying on their supervisors for learning [16]. Feedback and guidance provided by the supervisor determine the effectiveness of their supervisory relationship.

### Participants and procedure

The participants were selected based on ‘rules of thumb’ [17], and ‘information power’ [18], of the sample, using maximum variation purposive sampling technique [15], to get a diverse range of perspectives. The dimensions of variation were set to the participants' gender, area of specialty, current workstations [cities and hospitals] and place of training [local/foreign]. Associate Professors or Professors working in a clinical capacity, whether holding an additional administrative role or not, while also training post-graduate residents under their supervision were included in the study.

The data were collected between March, 2019 and August, 2019. An invitation letter to participate in the study comprising a brief introduction of the researcher and the purpose of the study was emailed to the participants a few days before the interviews. Out of the initially targeted 20, MS, who was an MHPE student at the time, conducted in-depth, semi-structured interviews with 16 senior clinicians. Of these, all except three interviews were conducted in faculty offices and were audiotaped using two separate devices. The other three were done over phone calls, using a free call recorder phone app to record proceedings. The duration of the interviews varied among different respondents but in general ranged from 35 min to one hour. All the interviews were later transcribed verbatim.

### Interview development

The interview guide was developed by themes derived from a thorough literature review and discussion amongst co-authors. Expert validation was acquired by a panel of four medical educationists. Furthermore, pilot interviews with two senior clinicians were conducted to assess and improve the duration, phrasing and flow of the planned interview.

The interview comprised engagement, exploratory and exit questions. The engagement questions, based on the leadership competencies by Fadil Citaku et al. [19], meant to help the participants relate these competencies to their own leadership experiences and answer subsequent questions accordingly. The open-ended exploratory questions allowed the participants the freedom to speak in detail in the manner of their choosing [20]. The exit question ensured that the participants could provide any other information that may have been missed out in the preceding questions. It was seen that the semi-structured interviews enabled us to extract information from the participants in the required depth.

### Data analysis

The authors used thematic content analysis to analyze the interview texts [21]. The data were managed via ATLAS.ti. The first two interviews were coded independently by MS and RAK, and differences in their coding schemes were resolved by discussion and consensus. Subsequent interviews were coded independently by MS who ran two coding cycles to derive themes to identify DAs and construct a framework for DACs. A second round of data analysis was done, and links were established between the DAs and the DACs that the study participants had identified based on their own experiences. A group discussion among the authors resulted in minor modifications in the framework. The final framework was approved by all authors.

### Quality assurance strategies

The quality assurance criteria of naturalistic studies outlined by Lincoln and Guba [22], was used for this study. Five peer debriefing sessions were carried out at multiple stages of the study [23]. Similarly, a reflective journal was maintained to note down the non-verbal cues of the participants during interviews as well as the researcher’s own biases. Participants were given interview transcripts via email for validation [24], and credibility [15].

The authors have analyzed the obtained data using the consolidated criteria for reporting qualitative research [COREQ] checklist described by Tong et al. [25]. The authors have also ensured data analysis triangulation by getting the data analysis verified with all authors at two points. Opinions of both majority and minority cases [that present ideas contrary to the majority opinion] were included in data analysis [15]. These strategies have been adopted to ensure the objectivity of the research findings [26].

Finally the authors defer to the readers’ own assessment regarding applicability of the study’s findings to different situations, while being hopeful that the study results will be reproducible in any similar future study [27].

## Results

Sixteen senior clinicians from nine teaching hospitals in five different cities of Pakistan, participated in the study (Table 1).

**Table 1:** Characteristics of participants

Dimensions		No. of participants [N]
Specialty	Medicine	3
	Surgery	4
	Gynecology	5
	Pediatrics	1
	Pathology	2
	Psychiatry	1
Gender	Male	6
	Female	10
Designation	Associate Professor	4
	Professor	12

### Developmental Assignments (DAs) for young clinicians

**Fig. 2:**
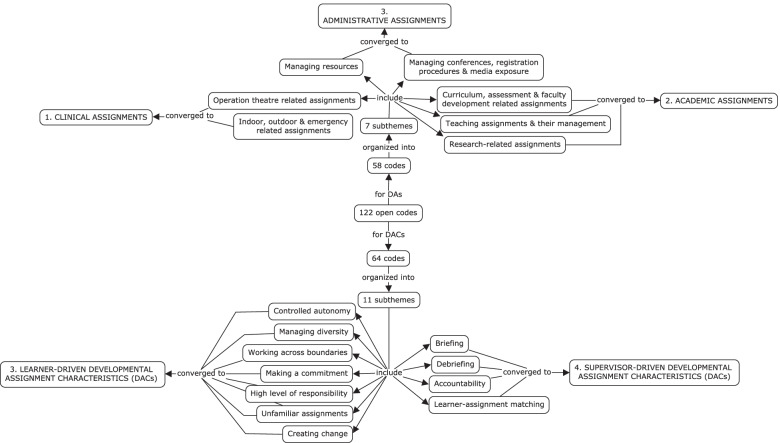
Detailed account of codes, subthemes and themes generated from data analysis

The data analysis generated 7 sub-themes for DAs which were synthesized into three themes: clinical assignments, academic assignments, and administrative assignments (Fig. 2).

#### Clinical assignments

Following and monitoring a surgical case, independent and emergency duties, counselling patients and their attendants, working with house-officers and nursing staff and working in infection control team of the hospital were considered developmental by the respondents.

#### Academic assignments

Academic presentations including grand rounds, morbidity mortality meetings and journal club contributed towards young clinicians’ leadership skills by polishing their communication skills. One respondent also emphasized on the importance of teaching different levels of students as well as patients.

*“I might be teaching the same topic to different level of students like it could be the postgraduates, the undergraduates or the nursing staff and if it is a disease, I could even be teaching my patients about it. So, my way of teaching will have to be different… That teaching would involve quite a lot of leadership or leadership like activities that would entail effective clinical teaching.”*

Other academic assignments that contributed towards leadership development were found to be related to conducting undergraduate exams, development of curriculum and faculty development programs, and research-related assignments. Regarding the latter, one respondent’s comment is noteworthy:

“When we were doing postgraduate training, we were asked to search ourselves and come up with a research topic…they did not spoon feed us. I feel that led to my growth academically and as a leader.”

#### Administrative assignments

In addition to clinical and academic assignments, the respondents described several administrative assignments that allowed leadership development. These include managing the coworkers and their roster, resource building, financial management of the department, developing SOPs for the hospital and managing the registration procedures of the department. Multiple respondents regarded managing an international conference as a trainee as developmental assignment for leadership skills:

*“We had an international conference in obstetrics and gynecology…I had to plan out and divide my time, make a timetable and then put the plans into practice… I think it helped me as it was a very high level of confidence as the outcome was well.”*

### Developmental assignment characteristics (DACs) for young clinicians

Related to the DACs, 11 sub-themes were found which were synthesized into two themes: Learner-driven DACs and Supervisor-driven DACs (Fig. 2).

#### Learner-driven DACs

The respondents recommended incorporating these DACs into clinical, academic, and administrative assignments for young clinicians to boost confidence and develop leadership skills in them [Table 2].

**Table 2:** Learner-driven and supervisor-driven developmental assignment characteristics (DACs)

S. No	DACs	Description
Learner-driven DACs		
1	Creating change	Create and facilitate change in a way the functions of healthcare system work
2	Controlled autonomy	Choice to make task-related decisions with pre-set informed boundaries
3	Unfamiliar responsibilities	Handling novel responsibilities
4	High level of responsibility	Performing activities with high personal or organizational impact
5	Making a commitment	Committing to provisional findings in written form
6	Managing diversity	Leading people from different cultures, gender, social and ethnic backgrounds
7	Working across boundaries	Influencing people or processes over which the young clinician has no direct authority
Supervisor-driven DACs		
1	Briefing	A session about task specifications and requirements before assigning the task
2	Debriefing	A two-way feedback session after the completion of the task
3	Accountability	Audit after the assignment is completed; involves accountability at all levels
4	Learner-assignment matching (LAM)	Task assignment after trait identification and providing support while maintaining the optimal level of challenge

The supervisors have to be mindful of patient safety in clinical assignments for ‘autonomy’ DAC, addressed in detail under the proposed LAM framework.

*“There are times when I need to understand and identify what it is that they can do, what is something in which they need to have my supervision. They should know when to call me.”* Encouraging the young doctors to develop research synopsis on their own was also found to be developmental in terms of leadership skills.

Participants also pointed out that young clinicians learned important leadership traits like communication and negotiation when their assignments required them to exercise authority. This led to two DACs; managing diversity, where a young clinician worked with diverse groups like students or patients on academic and clinical assignments respectively, where he/she has formal authority; and “working across boundaries”, where the young clinician had assignments requiring interaction with people and tasks outside his/her core area. A participant shared her own experience:

*“Where I was doing my postgraduate training…I had to repair the bladder so many times because the urologist was not available. That helped develop leadership skills in me.”*

Another DAC was having young clinicians “make a commitment” during relevant assignments like conducting morning rounds and living up to it. One participant explained how this was done in their department:

*“... when diagnosing patients, we encourage young clinicians to start writing whatever they see because when you write, you make a commitment...For future leaders, it is important to commit….”*

Additionally, “high level of responsibility” enabled young clinicians to gain further self-confidence and competence.

*“So, one way to develop leaders is that…you entrust them with responsibility early in their career. If you leave them till too late, then they will not change, they will not turn into leaders.”*

Some participants also pointed out the importance of fairness in such high visibility tasks to avoid a sense of favoritism among the residents. Here also, the participants advised giving patient safety the highest priority while adding this DAC to an assignment. The authors also found that the required developmental outcomes can be attained through assignments involving ‘creating change’ if the young clinicians’ resistance to change in some cases is addressed. This can be done if value of change is clearly communicated to the young clinicians and the change is introduced stepwise. Finally, the study showed that when a DA is unfamiliar to young clinicians and pushes them out of their comfort zones, it can help develop leadership qualities in them. The results showed that lessons learnt through one’s own initiative and effort have a deeper impact.

#### Supervisor-driven DACs

These DACS (Table 2) driven by the actions of the supervisor assigning the developmental assignment (DA) should also be incorporated into young clinicians’ job assignments to increase their developmental potential.

Data analysis revealed that correctly matching DAs to young clinicians based on challenge offered by the former and the competence level of the latter, had fundamental importance. The authors call this the learner assignment matching (LAM) DAC. In most cases, it was found that the best positioned to make this assessment for LAM were the supervisors.

The participants pointed out that it was important to structure the pre-task briefing correctly to allow it to play a role for leadership development rather than turn into micromanagement.

*“Briefing should focus on communicating quality standards, time standards and benefits of the task, not details on how to perform the task.”* Similarly a detailed two-way “debriefing” session after DA is completed was listed by the participants as a sometimes-overlooked DAC.

Finally, the participants highlighted the role of two-way “accountability” as a DAC. Accountability was found to make the young clinicians feel responsible and answerable. The two-way structure was found to help develop honesty and integrity.

### Relationship between DAs and DACs

The participant responses presented examples of routine job assignments performed by young clinicians along with the DACs that are applicable in transforming those specific job assignments into DAs. These findings were correlated by a second round of analysis to establish the relationship between DAs and DACs based on participants’ observations and experiences. Some examples of this relationship are given in Table 3. As an example, a supervisor, while assigning a young clinician to assist a senior during a surgery, can ensure leadership development in the young clinician by incorporating autonomy, high level of responsibility, unfamiliarity with the assignment, briefing, debriefing and accountability in the assignment, and adjusting the levels of these DACS based on learner-assignment matching.

**Table 3:** Relationship between DAs and DACs for leadership development in young clinicians

Developmental Assignments	Developmental Assignment Characteristics (DACs)
1. Clinical Assignments	
i. Operation theatre related assignments	
• Improving surgical techniques	DAC1, DAC2, DAC3, DAC5, DAC6, DAC7, DAC8, DAC9, DAC10, DAC11
• Assisting seniors during surgeries	DAC1, DAC5, DAC6, DAC8, DAC9, DAC10, DAC11
• Nursing the patient for the first 24 h after surgery	DAC1, DAC2, DAC5, DAC6, DAC8, DAC9, DAC10, DAC11
ii. Indoor, Outdoor and Emergency related assignments	
• Independent ward duties	DAC1, DAC2, DAC5, DAC8, DAC9, DAC10, DAC11
• Handling emergencies and accidental events	DAC1, DAC2, DAC3, DAC5, DAC6, DAC8, DAC9, DAC10, DAC11
• Managing patients in labor room	DAC1, DAC2, DAC5, DAC6, DAC8, DAC9, DAC10, DAC11
• Working in infection control team of the hospital	DAC1, DAC2, DAC3, DAC5, DAC6, DAC8, DAC9, DAC10, DAC11
• Counselling patients and their attendants	DAC1, DAC2, DAC4, DAC5, DAC6, DAC8, DAC9, DAC10, DAC11
• Patient diagnosis in outdoor or laboratory	DAC1, DAC2, DAC4, DAC5, DAC6, DAC8, DAC9, DAC10, DAC11
2. ACADEMIC ASSIGNMENTS	
i. Formal and informal teaching tasks and their management	
• Individual extempore academic presentations	DAC1, DAC5, DAC6, DAC8, DAC9, DAC10, DAC11
• Group presentations	DAC1, DAC2, DAC3, DAC5, DAC6, DAC8, DAC9, DAC10, DAC11
• Teaching undergraduate students, interns, junior trainees, paramedics, nursing staff and community	DAC1, DAC2, DAC5, DAC6, DAC8, DAC9, DAC10, DAC11
• Conducting Clinico-pathological Conferences [CPCs]	DAC1, DAC2, DAC3, DAC5, DAC6, DAC8, DAC9, DAC10, DAC11
• Participating in morning meetings and rounds	DAC1, DAC4, DAC5, DAC6, DAC8, DAC9, DAC10, DAC11
• Mentoring of students	DAC1, DAC2, DAC5, DAC6, DAC8, DAC9, DAC10, DAC11
• Managing exams	DAC1, DAC2, DAC3, DAC5, DAC6, DAC8, DAC9, DAC10, DAC11
• Managing LHV [Lady Health Visitor], nursing and paramedics training programs	DAC1, DAC2, DAC3, DAC5, DAC6, DAC8, DAC9, DAC10, DAC11
• Managing schedules for professor	DAC1, DAC3, DAC5, DAC6, DAC8, DAC9, DAC10, DAC11
ii. Curriculum, assessment, and faculty development related assignments	
• Involvement in curriculum development and management	DAC1, DAC2, DAC3, DAC5, DAC6, DAC7, DAC8, DAC9, DAC10, DAC11
• Developing assessment plan	DAC1, DAC2, DAC3, DAC5, DAC6, DAC7, DAC8, DAC9, DAC10, DAC11
• Making faculty development plan	DAC1, DAC2, DAC3, DAC5, DAC6, DAC7, DAC8, DAC9, DAC10, DAC11
iii. Research-related assignments	
• Developing synopsis on their own	DAC1, DAC5, DAC6, DAC7, DAC8, DAC9, DAC10, DAC11
• Helping junior trainees develop their synopsis	DAC1, DAC2, DAC5, DAC6, DAC8, DAC9, DAC10, DAC11
• Undertaking a research project and getting it published	DAC1, DAC2, DAC5, DAC6, DAC7, DAC8, DAC9, DAC10, DAC11
3. ADMINISTRATIVE ASSIGNMENTS	
i. Managing human or financial resources	
• Managing interns’ and junior trainees’ roster	DAC1, DAC2, DAC5, DAC6, DAC8, DAC9, DAC10, DAC11
• Managing the batch as batch in-charge, with inter- and intra-departmental communication	DAC1, DAC2, DAC3, DAC4, DAC5, DAC6, DAC8, DAC9, DAC10, DAC11
• Developing SOPs [Standard Operating Procedures] for the hospital	DAC1, DAC2, DAC5, DAC6, DAC8, DAC9, DAC10, DAC11
• Designing theatres, wards, ICUs [Intensive Care Units], dealing with stakeholders	DAC1, DAC2, DAC3, DAC5, DAC6, DAC7, DAC8, DAC9, DAC10, DAC11
• Managing a staff vacuum	DAC1, DAC2, DAC5, DAC6, DAC8, DAC9, DAC10, DAC11
• Managing financial resources for the department	DAC1, DAC3, DAC5, DAC6, DAC8, DAC9, DAC10, DAC11
ii. Managing conferences, registration procedures and media exposure	
• Managing an international conference	DAC1, DAC2, DAC3, DAC5, DAC6, DAC7, DAC8, DAC9, DAC10, DAC11
• Handling ISO certification, PM&DC and HEC recognition	DAC1, DAC2, DAC3, DAC5, DAC6, DAC8, DAC9, DAC10, DAC11
• Participation in live TV and radio health programs	DAC1, DAC2, DAC3, DAC5, DAC6, DAC8, DAC9, DAC10, DAC11
• Journal management	DAC1, DAC2, DAC3, DAC6, DAC7, DAC8, DAC9, DAC10, DAC11
• Laboratory management	DAC1, DAC2, DAC3, DAC5, DAC6, DAC8, DAC9, DAC10, DAC11

DAC1: Autonomy, DAC2: Managing diversity, DAC3: Working across boundaries, DAC4: Making a commitment, DAC5: High level of responsibility, DAC6: Unfamiliar assignment, DAC7: Creating change, DAC8: Briefing, DAC9: Debriefing, DAC10: Accountability, DAC11: Learner-assignment matching

### Framework for designing developmental assignments for young clinicians

**Fig. 3:**
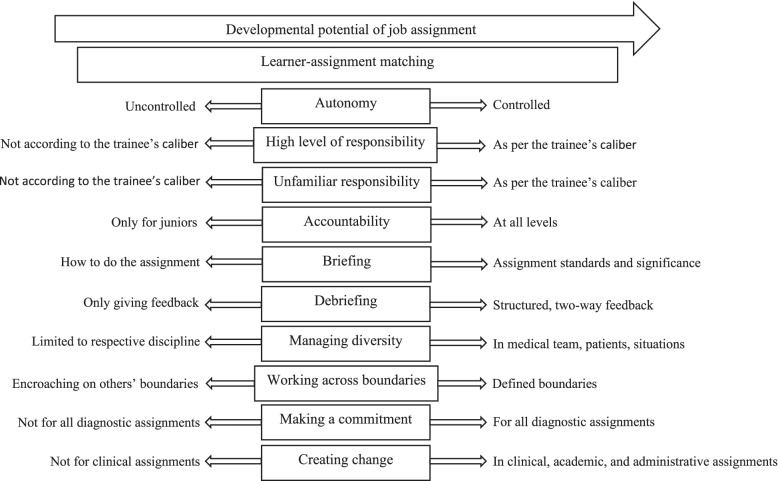
LAM Framework for designing DAs for young clinicians

The authors propose a framework for designing DAs (Fig. 3) based on this study’s results. This framework enables supervisors to configure relevant DACs for different DAs based on the learner-assignment matching (LAM) DAC described earlier. For making this adjustment, the framework here places the intensity of each developmental assignment characteristic on a slider scale. Using LAM, the supervisor can assess the match between a given young clinician’s competence [i.e., factor 1] and the level of challenge offered by the DA [i.e., factor 2] while using patient safety [i.e., factor 3] as a limit. She can then select the applicable DAC’s strength from a spectrum as opposed to a binary present/absent value.

As an example, the first DAC shown here is ‘Autonomy’, which has values ranging from ‘Uncontrolled’ to ‘Controlled’. The more the DAC level is set towards the left on this spectrum for a DA, the lesser the autonomy the young clinician undertaking that DA would have. This could happen when the supervisor finds the challenge offered by the task to exceed the young clinician’s competence, or when patient safety concerns act as a limiting factor. The more the DAC values move leftwards on the spectrum, the lesser the developmental potential of that DA. Still, LAM guidelines and patient safety are important to bear in mind while setting the value of a DAC in a DA. Patient safety has paramount importance in the healthcare domain. This process applies for all DACs in general.

This framework can be used by clinical supervisors to design developmental assignments for young clinicians in their own context to extrapolate the study results.

## Discussion

The aim of this study was to explore experience-driven leadership development in young clinicians in the specific context of a healthcare organization. Subsequently, the authors have proposed a framework that validates the five DACs for leadership development identified by McCauley [28], and Ohlott, [12], after converting them from having binary values to being on a spectrum. Furthermore, they have proposed six additional DACs that allow this framework to be applicable specifically in healthcare organizations.

They key to using this framework is the proposed LAM DAC. It guides a supervisor in adjusting the level of other applicable DACs in a DA based on two factors namely learner’s capability and task challenge, while being constrained by a third limiting factor namely patient safety. The learner capability factor is supported by Savage et al. [29], who confirm the value of assessing the learner’s capability and matching the provided development opportunities with that. The task challenge factor is supported by McCall [30], and Ohlott [31], who consider the level of challenge offered by the task to be a key driver in developing leadership skills. The proposed limiting factor for LAM is patient safety, which is confirmed by Stoller, [32], who describes it as a necessary constraint in leadership development in clinicians in healthcare systems.

The first two of the DACs from Ohlott, [12], the authors have also found applicable for young clinicians, are the role of autonomy, and the level of responsibility in a DA. The role of autonomy here resonates with the educational implications of constructivist theories [33], and andragogy [34], and is also discussed by Andersson [35], in terms of leader identity development. According to him, physician autonomy during their clinical assignments allows physicians to develop a belief in themselves and hence, develop as leaders. Moreover, a recent study examining university faculty revealed greater autonomy as a positive factor in enhancing leadership development, [36]. Similarly, the authors found that incorporating a high level of responsibility in DAs is linked with high personal or organizational impact and serves as a DAC. An important challenge related to both the autonomy and high responsibility DACs is to keep a perception among the young clinicians about fairness of opportunity. This is confirmed by Roger Kline [37], that if fairness is not seen, it results in increased competition and decreased cooperation at the cost of teamwork.

Also in line with Ohlott, [12], the current study confirmed that creating change acts as a DAC that enables leadership development especially when young clinicians understood the value of this change in a DA. These findings are confirmed by Savage et al. [29], who identified the foremost importance of explaining the rationale for change to one’s subordinates. The current study also discovered a lack of such assignments currently for young clinicians at Pakistani teaching hospitals in part due to concerns amongst senior clinicians for patient safety. This problem can be addressed by having the supervisors control perceived risk via the patient safety factor in LAM.

Furthermore, the degree to which young clinicians must work across the boundaries of their job description and manage the diversity that comes with interacting and working with diverse people are two DACs the present study has confirmed, synonymous with Ohlott’s findings in the corporate sector, [13].

The authors now proceed to a discussion of the additional DACs proposed by this study. Pre-task briefing is reported as a DAC that leads to leadership development by allowing a supervisor to maintain the DA’s challenge and complexity within the desirable range. This balance is important; Dóci & Hofmans [38], describe a negative association between overwhelming assignment complexity and leadership development. Another study on organizational leadership development [39], showed that the developmental value of an experience reaches a point of diminishing returns beyond a certain level of challenge.

The current study has proposed visible accountability at all levels of the organization as another DAC. This role of bilateral accountability in leadership development is also supported by McCall, [40], who demonstrates accountability to be an important attribute of an effective leader in corporate organizations, while McKimm & Swanwick, [41] show this for modern healthcare organizations.

Another DAC proposed by this study is getting young clinicians committed to the diagnosis they make. This helps them learn integrity and self-confidence. These are essential leadership attributes for healthcare professionals, [29].

Finally, the present study showed that another important DAC is debriefing in the form of a two-way feedback after the assignment finishes. Zaccaro and Banks, recommend a similar approach as it activates self-regulation mechanisms and encourages the young clinicians to reflect on their learning by the assignment. The importance of this is also confirmed by several experiential learning theories such as those proposed by Dewey [1938] and Kolb [1984], [42].

The authors hope that this study’s approach of converting existing job assignments to DAs for leadership development will allow senior clinicians in diverse settings to utilize these results in their respective settings. And since this approach builds on top of existing young clinician job assignments, it has the advantage of not creating any extra load on their schedules.

### Limitations of the study

The authors note some limitations in this study. First, the study employed an exploratory methodology to focus on perspectives of senior clinicians only. Focus group discussions of current young clinicians could have been helpful in providing their perspective. Second, while the present study shows how developmental challenge in one’s job experiences is an important determinant of leadership development, it does not address how developmental work experiences should be sequenced to optimize leadership development.

### Strengths of the study

Strengths of the study include its multi-center design; anonymity and confidentiality of responses; use of ‘information power’ of the sample to determine the sample size; inclusion of participants with diverse public and private post-graduate training experience geographically distributed across the country and the use of semi-structured interviews to obtain in-depth information from the participants. This study has contributed to the existing literature by providing an action-able framework for designing developmental assignments (DAs) for leadership development in healthcare sector.

## Conclusion

Today’s healthcare professionals and in particular clinicians need to be equipped with leadership skills along with clinical skills. The most effective way to teach these is via experiential learning. Models for this exist in the corporate sector, but the specific needs of the healthcare sector dictate that those methods cannot directly be employed here. This study builds on studies in the corporate sector to propose a model for experiential leadership development in the healthcare sector. It identifies 11 developmental assignment characteristics (DACs) that can be added to regular young clinician job assignments to elevate those assignments into leadership developmental assignments [DAs]. This study also proposes a framework based on learner-assignment matching (LAM) for supervisors to set the appropriate level of these DACs in DAs. This LAM factor is driven by the capability of the learner and the challenge offered by the assignment and is constrained by patient safety. The authors hope relevant decision-makers in our healthcare education infrastructure will find this study practically helpful.

### Future research

An interesting angle to explore in future studies could be the perspectives of young clinicians. It might also be interesting to study the effect of DAs on leadership development in young clinicians in an interventional format. Another interesting research would be to study which leadership competencies are correlated with the different DACs proposed in this study. Future research could also consider other learning outcomes through experience-driven leadership development. An example could be studying how the young clinicians’ leader identity, professionalism or clinical competencies are shaped by developmental experiences.

## Supplementary Information

**Additional file 1.** Interview transcriptions. The additional file 1 contains anonymously coded and transcribed qualitative interviews from the study participants.

## Data Availability

The dataset supporting the conclusions of this article is included within the article [and its additional file].
