# Supplementary material for: Exploring developmental assignments shaping experience-driven acquisition of leadership competencies in young clinicians
Source: BMC Med Educ. 2022 Jun 28;22:505. doi: 10.1186/s12909-022-03544-y (PMC9238052; doi:10.1186/s12909-022-03544-y)
Supplement: Supplementary file 1 — Additional file 1. Interview transcriptions. The additional file 1 contains anonymously coded and transcribed qualitative interviews from the study participants. [file 12909_2022_3544_MOESM1_ESM.docx]

**Developmental assignments shaping experience-driven acquisition of leadership competencies in clinicians.**

**Interview from R1**

**Duration: 60 min**

**Q: What do you understand by leadership?**

Leadership, in its literal sense, involves someone who leads. Someone who leads can be a leader of a pack, leader of a school, leader of a college or university, someone who is in a leading position and is leading people who do not have the experience of coming up to the mark of the job that they have been assigned. So in my dictionary a leader is an orchestra controller who is able to use the people with him to the best of their potential, be it an organization or an orchestra.

**Q: What, in your opinion, are the leadership qualities that you rely on at your workplace?**

I think if you ask different people the same question, everybody will obviously give you different answer. I think this is the reason you asked me my view about it. I think the way I conceive a leader is someone who has to be honest, dedicated and all those qualities that I noticed from you list sent to me. No one person can have all those qualities. You can have some of the qualities or many of the qualities but obviously you would be an extraordinary gifted saint if you possessed all these qualities. So it is not humanly possible to possess all those listed qualities. So what is the best? You must have heard something about this and it holds true for my dictionary that “You are either a leader or you are not” and that “You are a born leader”. So I think someone who has the capacity to lead. That is something that does not come with assignments. The assignments cannot inculcate in you the personality to lead. Few words that I use are you should have the drive to kill, you must have a certain frame of mind, a certain mental strength, a certain IQ level. If you are trying to give on-the-job assignments and trying to create a leader by way of workshops, by way of telling them how to climb the ropes of leadership, by trying to bring them into the sphere of leadership by telling them how to behave in a peer relationship, how to work in group dynamics, these would all be excellent tools for somebody who is a born leader and has the potential. Somebody who is a PhD and is a very knowledgeable person but does not have the personality, no matter you spend X number of assignments, X number of months on them, they would not be able to lead. So to be able to lead is a quality. All those qualities that you listed are ideal but if you identify a person with leadership traits and if you train that person, then he/she will turn out to be an excellent leader in a global/general sense. You can identify a raw talent in a student or a house officer or a young doctor. If a talent is spotted by a senior person or a teacher or a mentor who is on the lookout for training people for leadership qualities. A leader can be good or bad. Hitler was a bad leader. Ataturk was another leader. Jinnah was a leader. Gandhi was a leader. Nehru was a leader. Mujeeb-ur-Rahman was a leader. Nelson Mandela was a leader. There are people in the history who were leaders. Even in Mughal Empire, there were six Mughal Badshahs. Three of them were great leaders, two of them were weak leaders, one doesn’t carry much weight in history. So this is what I am trying to say. If you have an opportunity in an organization where you want to develop leaders and that organization houses students and young doctors then somebody should be on a lookout to identify people who they think have the potential and then incorporate that concept of your assignment-based leadership qualities development. They would turn out to be better leaders. The assignments should be tailored according to the potential of the student or house-officer. Our job should be to identify the potential. That’s what we do for nomination of CR and GR. When the students come to us that we have applied for CR and GR, we don’t appoint anyone because we want to appoint the students with leadership potential and then we groom them.

**Q: Literature says that 70% of leadership is developed by developmental assignments (on-the-job tasks). What developmental assignments can you think of, that you would credit, from which you acquired the aforementioned leadership qualities?**

I think during my career, the thing that helped me develop leadership qualities was the type of job that I do. Professionally, I am a cardiovascular and thoracic surgeon. So by default when I came back to this country in 1995, there wasn’t a great infrastructure or environment for cardiothoracic surgery in this country. I had to start this specialty in places where it did not exist. For example, I started operating in XYZ center where the facility just did not exist there. So for me to be able to start the facility I had to do everything right from the scratch, from creating designs to creating theatres, wards, ICUs, recruiting lower staff, paramedical staff, doctors and train them all and then do the first case and then sit with the case for the next 24 hours and nurse them as well. So the nature of my job since the beginning involved leading all those people; architects, paramedical staff, administration, finance. My exposure to administration was quite early. So one way to develop leaders is that you identify people if you have the infrastructure and you entrust them with responsibility early in their career. If you leave them till too late, then they will not change, they will not turn into leaders. Let me give you an example. If there is a CR or GR or a PG with extraordinary potential and you entrust him/her with certain leadership responsibilities in the form of on-the-job assignments then those people may turn from Bahadur Shah Zafar to Babar. Some people will learn on the job like I did. But maybe my structured training in UK as behind this. I went through a structured training program and when I had to do it here, I replicated that here. Identify a person with the potential and then throw him in the deep end with the training.

**Q: If we dig a level deeper, what was it about these experiences that made them a positive learning experience?**

I think you need to have courage. For example, you are assigned a task according to rules and regulations. There are some decisions that you may have to take outside of these rules but that would involve a lot of common sense. Because I believe that if you read the book of law, it is nothing but common sense. If it makes sense to make a decision and if it is not defined, then you should take it. That is what I mean by courage. You have to think outside the box. Being a leader, you have to go outside the defined boundaries as well and that is why I used the word that you have got to have courage to be able to do that. For example being a pro-rector here I have a lot of scope to do things here but sometimes I do things which make sense for me to do but I am not expected to do them but if you do them and if your calculation is right and it ends up well then it adds to your profile. I believe a leader creates his own profile, he creates his own job description. He can be a very safe leader by not going outside his job description and nobody can point a finger at him or he can be a little courageous in going a little outside of his job description and do the right things and land success. You have to take risks, not with the patient’s health but in the organizational structure, for example, deciding whether you have to go for a traditional or integrated curriculum program. The way I replicated my specialty here was something that had never been done before, so it was risky. If it lands properly, it will bring you credit and success and will add to your competence and leadership qualities like I have been tested here as I have changes the entire education system of this medical college. If I wanted to play it safe and do my job within the defined parameters, the university authorities were not going to ask me why you are not changing. They never delegated me to change anything, yet I changed everything.

**Do you think your training in UK develop any leadership qualities in you?**

I think it certainly did. For example, I am not the only cardiac surgeon who returned to Pakistan in that period. There were other people who returned. But then I in Lahore am the person who has created about six centers where cardiac surgery is being practiced today. I created them, I was the person who did the first operation there. I created a facility and then I moved on to another center and I created another center. But there are people who returned with me from the same system never got the opportunity to do this because they found it easier to join a system which was already working. So you need to have that trait for leadership. If I get this opportunity now, if I go to the UK, US or the West in an organization and find Pakistani people in an area or a specialty and if I know that they want to come back and if I get the chance to spot them then I will spot them and come back and probably help them create something for them so that they don’t have to go through those hardships that I went through. So creating a whole system was the most important and the most difficult thing that helped me develop leadership qualities.

**Q: As a clinical leader, you assign tasks to you subordinates/juniors. Do you consider incorporating certain elements/characteristics in those tasks in order to help the assignees grow as leaders?**

I believe in delegation of responsibility to selected people. In my experience, random distribution of jobs does not necessarily translate into good outcomes. If you want good outcomes then you need to build up a proper stem or a questionnaire to be able to have the good answers from them. It is on-the-job assignments that will help create leaders but the material for that has to be right. You might need to compartmentalize the main job into many sections. You can take the example of you own MHPE program. I am a strong believer that these programs not only train but add to people’s personalities a lot. But yet in a class of 20 where everybody is from a different background, yet not everybody who qualifies goes out to practice. Only few of you will go out to practice MHPE once you have attained one. Why have the other people not decided to practice it? Maybe they just wanted to use it for some additional qualification or promotion. Similarly, if you just randomly assign the people different jobs and delegate them responsibilities in organizational setup, its translation into good outcomes is important. Another factor is the formation of regulatory bodies to monitor the outcomes. This was started in the West but has been adopted here as well. For example to regulate the practice of Medicine, you have either Medical Councils or Healthcare commissions. The need to develop these arose because uniformly everybody was not doing their jobs right. So to regulate them, they needed regulatory bodies all across the world. So relying on self-monitoring is not enough, a monitoring system has to be there for the job assignments as well to provide a check and balance system. Self-auditing is important so that you can develop as role-model.

Maintaining quality can be taught by tasks. The task will help them get to a different level altogether. The leadership qualities that can be acquired by developmental assignments Personal decision quality, maintaining safety, task knowledge, honesty and integrity. Furthermore, accountability can be taught by tasks by telling what you have done right and what you have done wrong. Time management and goal orientation are some things that can be taught by assignments. Effort to achieve goals and persistence despite challenges can be taught as the more you work over a task, the more you get better at it. Some people are good in seeking feedback and some are poor at it. This is a very important trait that is related to learning skill. Feedback by taking questionnaires after a task can incorporate this competency in the trainees. Communication with co-workers, active listening can be taught by group dynamics. Facilitating discussion can be taught by tasks if the tasks have room for discussion, if the tasks are related to performance only, then it won’t be possible. Taking charge and orienting others, developing and building teams can be taught by group-oriented tasks. Setting goals for others is subjective and can be taught by incorporating group dynamics in the task. Reinforcing success can also be taught by developmental assignments. The quality of debriefing after a task is very important in developing these leadership competencies as it is a form of feedback. Developing external contacts can be taught by community based assignments. Principles of learning can be taught by briefing before the task. Assessing others can also be taught by job assignments. You make them do a task and then let them assess others in the format of ‘See one, teach one”. Same goes for coaching and instructing. Empowering a trainee to do a task, for example, an appendectomy, all on his own will give him confidence. In clinical context, autonomy should be transferred in tiers. Limited autonomy is given at each tier. A house officer is not expected to do any practical procedure. He is supervised to be able to clerk right. Admit right and do some initial work up. Too soon an autonomy is not beneficial and complete autonomy should be given when you have completed all the learning tiers. Critical thinking, creative problem-solving, identifying problems, seeking improvement,

Legal regulations, open door policy, servant leadership cannot be taught by job tasks. Servant leadership is a quality and a servant leader is someone who I can call a passive leader who leads by example. You can get people to copy this behavior but not by tasks. Distributing rewards fairly, financial ethics, taking initiatives. Self-control, self-tolerance and adaptability are more of personality traits that can be judged better by others. Somebody will need to tell you that you have this deficiency, you need to cool down, and you need to calm down. These are acquired tastes. So, in a sense, they can also be taught by assignments but somebody will need to bring your attention to it. Learning Psychological knowledge requires wide exposure. This is a very difficult thing to develop. You cannot really accomplish that with task only. Social perceptiveness and nurturing relationships cannot be taught by developing assignments. Cooperating is the backbone of group dynamics and is a personality trait. Same holds true for resolving conflicts and negotiating. Political awareness does not have a direct relationship with job tasks. Openness to ideas, collaborating are very important but are is again personality traits. Perceiving systems is also not task-related. Evaluating consequences, visioning, managing the future, sensitivity to situations, challenging the status quo, intelligent risk-taking, reinforcing change are important leadership qualities but cannot be related to the task.

­­­

**Developmental assignments shaping experience-driven acquisition of leadership competencies in clinicians.**

**Interview from R2**

**Q: What do you understand by leadership?**

Leadership is leading people and taking them along with you.

**Q: What, in your opinion, are the leadership qualities in you that you rely on at your workplace?**

I think understanding the needs of the people and accommodating and listening to them, respecting their opinion.

**Q: Literature says that 70% of leadership is developed by developmental assignments (on-the-job tasks). What developmental assignments can you think of, that you would credit, from which you acquired the aforementioned leadership qualities?**

Managing the Rota, I was in-charge of my batch and then I had to guide that batch during that duty or rotation and take it forward. I was assigned these duties and I was held accountable for them later on, helping in developing leadership skills.

In Gynecology department, the patients in labor room are my responsibility. If something happens to any patient, I will be asked about that. If you are given responsibility about the management of a patient and the next day you are asked about it, then it is useful. Responsibility should be given to the trainees to help them develop as leaders.

Level of autonomy: You can’t give a higher level task to a trainee, you can’t jump these steps. If you give a higher level responsibility to a trainee, the other develop a political scenario out of it and call it favoritism. So it is better to distribute tasks according to the pre-defined levels. But sometimes you have to task the trainees at a higher level depending on the situation.

We were sent for academic tasks as PG trainees for example, teaching the paramedics and nurses. This taught us how to lead a class, how to interact with them.

**Q: If we dig a level deeper, what was it about these experiences that made them a positive learning experience?**

Role-modelling is very important and counts a lot. Moreover, opportunities should be given to the trainees. Sometimes we work in such groups where one senior says that all the tasks will be done by him/her. There is no task delegation. In such a case, the trainees or juniors cannot grow as leaders.

Q: What, in your view, is the effect of Briefing before assigning a task & Debriefing after the completion of the task on leadership development of the trainee performing the task?

Yes, instead of expecting him to blindly go about the task, if you give the person some orientation before a task that will be better for him/her.

Debriefing is also very important because if you do not get a feedback, you will not realize your mistakes and your problems will keep growing. You do learn from personal experience and hit and trial but if you are getting a proper feedback from someone, then that will definitely enhance your leadership skills.

**Q: As a clinical leader, you assign tasks to you subordinates/juniors. Do you consider incorporating certain elements/characteristics in those tasks in order to help the assignees grow as leaders?**

I send my trainees for teaching assignments to take third year classes. I inform them beforehand and brief them about the task. Once they are done with it, I take their feedback about their experience. This gives them confidence and boosts their leadership skills.

**Developmental assignments shaping experience-driven acquisition of leadership competencies in clinicians.**

**Interview from R3**

**Q: What do you understand by leadership?**

The person who is called a leader should qualify for those qualities, he should have some inspirational personality and he should have the ability to encourage and intrinsically motivate the followers/ students or employees.

**Q: What, in your opinion, are the leadership qualities in you that you rely on at your workplace?**

I think the first and foremost quality that I have to employ at my workplace is tolerance. If I am not tolerant towards the mistakes of my juniors, I will create a havoc at my workplace. Then I think adequate background knowledge about my subject is very important, a certain level of skills is required because if I do not have those skills, my juniors will not follow me properly. Attitude is extremely important and then motivational skills again. First of all, I should have an internal drive and I should personify it. I should not have to say things to my juniors, they should rather try to follow me. That is what I try to do at my workplace. I do not know if I am a hundred percent successful in that.

**Q: Literature says that 70% of leadership is developed by developmental assignments (on-the-job tasks). What developmental assignments can you think of, that you would credit, from which you acquired the aforementioned leadership qualities?**

First thing I remember in my house job was that my seniors asked me to do a PowerPoint presentation which was quite rare in those days and I was scared that how I would do it. I did it and it was very successful and it created a confidence in me that I can do it. Then there was another incidence in house job when they asked me to do the stitching and it was compared with my senior’s and it was appreciated. This gave me confidence that I can do better than my seniors. In FCPS training, there were so many assignments that were given and I completed them successfully like doing certain tasks and courses, assisting my seniors.

I have managed rosters as an associate professor and currently, I am doing the job of director of labor room where I have to manage the nurses, the paramedical staff, the house officers of three units. So that is the most difficult thing that I am doing and alhamdulillah I think that I am successful.

I take classes of nurses and it has helped me in developing my leadership skills. When you go and you communicate with nurses and you identify gaps in their knowledge and their problems and then you try to help them solve them, in this way you come to understand their psychology and you develop some sort of sympathy with them also that what are their issues and how can they be incorporated to develop a better clinical team, because ultimately in all clinical setups, it is the team work which matters.

**Q: If we dig a level deeper, what was it about these experiences that made them a positive learning experience?**

Q: High levels of responsibility: Do you think that high level of responsibility in a task can help in developing leadership skills in the person performing the task? Also, how does this occur and can you relate to it in the clinical scenario?

I think that is very important. If you are given a high level of responsibility and you do it well, then you earn the appreciation of your seniors, your peers, your colleagues. You earn respect and your own self-confidence increases with that.

Q: What do you think should the level of autonomy while assigning a task?

It should be supervised for the trainees. We are working in very high-risk situations, so we cannot give them full-fledged autonomy during training. After training at senior registrar level, that is possible but at the trainee level, they should be supervised.

Q: If you feel that a first year resident has the caliber of performing a second or a third year resident’s tasks and you feel that doing so will enhance leadership development in that trainee, will you assign a higher level task to him/her?

I would like to give such a person a chance but in practical situations, things are different. If there is a good worker, I think that worker should be encouraged but the thing is that there is too much competition at the level of the trainees. If you give extra tasks to one trainee, the other 18 or 19 trainees will start weeping. So practically it is not feasible. You can maybe give him/her a few extra tasks because it is always more rewarding to work with an energetic worker.

**Developmental assignments shaping experience-driven acquisition of leadership competencies in clinicians.**

**Interview Transcript from R4**

**Q. What do you understand by leadership?**

I think that leadership is the quality in a person to handle his/her own responsibilities and also to give the right direction to the people working under her and also make sure that the things being done by her and by her juniors are safe and not hazardous to them and to others whom they are dealing with and they are able to look into the details of the task, like what they are doing and how it will help them, how it will affect them in the future, what are the benefits that they are going to get in the future by doing this task at this time.

**Q: What, in your view, are the most important leadership qualities that you rely on at your workplace?**

I think I am quite observant and I usually give time to the juniors to do whatever they want to do so that they are able to express their potentials and only after they have performed, I guide them that what was right and what was wrong and what could be improved and how they should go further next time with this type of task.

**Q: Literature says that 70% of leadership is developed by developmental assignments (on-the-job tasks). What developmental assignments can you think of, that you would credit, from which you acquired the aforementioned leadership qualities?**

The foremost thing is decision making while you are on duty in the emergency. I think I was always given free hand to decide first and them consult the seniors that whether my decision is right or I have to change it to some extent. So increased level of autonomy helps in developing leadership qualities.

The other thing is whatever is being done at the floor, I was not forced to hide anything, like something will happen and the seniors will get angry with me or complication has occurred and they will snub me, it was not like that. I was feeling safe enough that I can tell them and they will be helping me instead of just suppressing me. So this was a very important factor.

The third thing was the equal distribution of opportunities. In my training, I got a large number of opportunities and it was never like I had to rush for something or ask for some procedure or to get any favor from someone. I got most of things by turn or by right. So this was not the stress element for me that my training is ending and I have not done this or that thing and I need to do this, so I have to ask that person and whether that specific person will favor me and I will get that procedure. I was never like that. I was comfortable while doing training and that gave me peace of mind and even after training, I feel that this is the major factor that still I am working in Gynecology department and most of the times people say to me that you don’t behave like a gynecologist. This is the reason behind this that I was never threatened by the immediate on-call seniors and I was free to make a decision about the patient and implement it and everyone was willing to share even the good and bad consequences with me.

I was in-charge of managing the class lectures of final year for the professor. So I was making the schedule and managing the timings and I was responsible for informing the person who was going to take the lecture so that they are ready in time for that and don’t miss that and also the class demonstrations that were being done by us in the department, I was preparing them and presenting them. So this was improving my confidence level and my presentation skills, communication skills and also the ability to answer questions, take questions, that as quite helpful. Also while preparing for the exam you were doing all the practice, like of exam pattern and everything was rehearsed in the same environment. We were doing that practice again and again. We were asked and them we had to justify our answer, we had to explain. We had to either commit to them or prove ourselves right. So we were rather made adjusted to the type of environment that we should listen to others as well and we should also stress on our points as well. If we think we are right, we should give proper reference for it so that the other person is convinced.

The managerial and academic tasks also helped me in developing leadership skills. All these exercises help in the clinical scenarios a lot because clear mind and ability to listen to others, both juniors and seniors, especially when you are doing a surgery, you have to listen to the staff and nurses, the OT Attendants. Sometimes they are pointing towards the very right thing that you are missing. So while you are working in a team you have to listen to everybody but you have to give weightage to the thing that is important, not to everything. So these exercises in practical scenario were very helpful because when you go in the practical scenario and once you are a leader performing a task, everyone is under you, they will be helping you, but they will be looking towards you as well so that you can give them the proper direction, the proper pathway to move forward and also to act in a safe manner. So I feel that all these exercises helped me a lot while working independently in a unit.

**Q: If we dig a level deeper, what was it about these experiences that made them a positive learning experience?**

I feel that during training, I was a PGR. I feel very lucky that I did training in Gynecology, even though I never liked Gynecology very much because I found it to be too hectic, disturbing and dirty. I was not ready to work in the labor room while I was doing house job in surgery department. But my friends were doing Gynecology, so naturally social pressure and societal pressure forced me into Gynecology. Actually I was lucky enough that when training started I just worked under some immediate senior for two to three months. Then our batch was comprised of 2 people of equal level: one me and one another doctor who was a graduate of FJMC. We were both at equal level and we were doing surgery and the senior registrars were very helpful and trusting, they were teaching us as well. So we got the opportunity to do a lot of things that people don’t usually get even after one and a half years. So after six months of training, I had done a hundred cesarean sections. So that gave me a lot of confidence. It was not just cesarean sections, but naturally I was managing all those patients at the same time and I was given opportunity and trust that I can do more and that I should do more. So that helped me a lot. This is the reason I feel that people should be given opportunities. When we give them opportunities, they usually perform well. Whenever you feel that it is becoming risky and unsafe, you can stop them but opportunities should be given so that they can act in a better way and they can use their own mind and abilities to perform the task.

**Q: Certain characteristics of tasks that help in leadership development have been identified in the literature. Can you please elaborate on and relate to them in clinical tasks?**

High levels of responsibility: When you give responsibility to a certain person and you have said that that person has to perform this and will perform in a specific time frame and will be of a specific quality, the person feels that he/she is responsible and answerable for that. There must be a certain incentive attached to the completion of the task in any form, some reward, some certificate, even applause. That will be very beneficial, so assigning responsibility is always developmental. We should not go out of limits, we are in limits but we are starting from the basic and increasing the level gradually. We are going to a certain level. Naturally if a person is a house officer, we will not give him/her the assignments of a third or a fourth year because that will be dangerous. However if I feel that a person has a caliber, I will give him/her a chance, not of a much higher level. But if a house officer is of a higher caliber, I feel that some more chances or a higher level of responsibility should be given to him/her, I will give him/her a first year resident’s level of responsibility. That will improve and polish her qualities.

Creating change: I think that we should create change but it should not be just for the sake of creating change, it should be for the betterment and it should be stepwise. We should acclimatize the people involved in the change. We should make them agree with the change and then go ahead with it instead of just ordering it. We should discuss with the people, its benefits, hazards, long term effects and how the world is developing and how this change will help us to meet those levels of development. Such a change will be beneficial, not just an abrupt change of system or routine.

The change in roster is also an example of such a change in clinical system. Such a situation happened in my department a few days back and there was a lot of protest from the residents. The roster was changed due to a shortage of residents and there was a lot of hue and cry but then I asked the senior registrar to sit with them and tell them that whatever the situation is, we are going with that. So if the PG trainees are short, we cannot skip a day, we had to make a roster and naturally you will get more duties and more opportunities. So I think while you are doing your duties, you should be sincere with your training. You have to adjust according to the circumstances.

Getting the trainees involved in academic tasks like curriculum development, management and assessment will also help them in developing their leadership skills because this will help them in getting a know-how of basic things that they are going to deal with in the future. They will be more efficient in performing those tasks in later time.

Unfamiliar responsibilities: They will be able to handle the situations that they were not familiar with. They will be all alone to deal with that situation. That will give them two options: either that person will collapse or he/she will deal with it. If they deal with it, even to some extent, either partially or completely, that will boost their confidence. That will open their minds to look for what they have done and what they have missed that they should have done so that they can improve for the next time. So these things are always learning experiences.

Working across boundaries: Yes, for example people go for LOCUM or during training with CPSP, they go abroad for a couple of years, is very helpful for leadership development because that will open the horizons and they will be able to know what people are doing and what we are doing and how we are lacking behind or how we are good at certain things and how can we implement those rules, regulations and policies in our setup, and they will be able to understand how the other policies and systems are working and how our policies and systems are working.

Managing diversity: When you are making a team, a leader should listen to everyone, every type of person at every level, giving respect to them and explaining his/her own point of view in a decent way so that they can easily communicate with each other and can exchange their ideas to reach a final conclusive plan of action. A team should be diverse and everyone should be tolerant enough, only then a team can work. If a team members are not tolerant towards each other, the team cannot work and the results will be definitely poor.

**Q: As a clinical leader, you assign tasks to you subordinates/juniors. Do you consider incorporating certain elements/characteristics in those tasks in order to help the assignees grow as leaders?**

I think if I am able to make it interesting and I am able to convince them that it will be beneficial for them in the present as well as in the long run, then they will be more interested in doing that task. If we turn that task into a competition, for example, between the house officers, or senior residents amongst themselves, they will be more enthusiastic in performing that task and they will try their best.

If I am telling them what went well, what didn’t go well, how the task can be performed better and what were the mistakes that can be corrected and how can the outcome be improved, only then they will be able to realize that this is an important thing and they will be able to practice this in the future, because if I just pass an order and say that everything was good or everything was a disaster, they will also develop the same type of habits. So that is very important that we first brief them, then evaluate the performance and finally debrief them. Debriefing is very important because only then they can be able to realize what their deficiencies were and how they can improve them.

**Developmental assignments shaping experience-driven acquisition of leadership competencies in clinicians.**

**Interview from R5**

**Q: What do you understand by leadership?**

When you are given a task in a profession while you are doing teamwork and you are given the responsibility of every person in that group, you have got to set down SOPs regarding your work distribution and you have to watch out that all the persons are trained enough and skilled enough to carry out the responsibilities that you have given them and to keep an audit or feedback on their performance. You also have to encourage them to improve whether the improvement comes from you or you have to get help from other sources. You have to develop contacts for that and you have got to keep your people on the look. That’s what I understand by leadership.

**Q: What, in your opinion, are the leadership qualities in you that you rely on at your workplace?**

At the moment, I feel that I am a good listener. I listen to everybody’s problems, everybody’s suggestions and according to their personality prototype, I try and mold them. Their suggestions could be valid or invalid but I am not there to offend them or given any derogatory remarks to them which is what we have suffered from our seniors. I don’t want to repeat the same mistakes. I want to be a more positive person in my environment. I think this is what counts me out of all the other people which I think is my quality is that I want to remain positive no matter what is happening - Best performance in the worst circumstances.

**Q: Literature says that 70% of leadership is developed by developmental assignments (on-the-job tasks). What developmental assignments can you think of, that you would credit, from which you acquired the aforementioned leadership qualities?**

Learning new skills as science is always progressing helped me in developing leadership skills. Also, I helped a number of my trainees develop their synopsis and dissertations, we got two research projects. That was a new start here doing the fetal blood scalp sampling and doing colposcopy and Alhamdulillah I have been running the colposcopy department for the past ten years. So yes, I can say that I have been a part of those challenging assignments. I faced a number of problems especially starting the thing was very difficult and once it was started, maintaining it was a challenge. All the resources, the resource building, the finance, the human resource, it was all very difficult. The everyday challenges that I have, I am still carrying on with them and I think most of my targets are alhamdulillah still in action. The ups and downs are always there. If one is happy with the ups, one should be prepared to deal with the downs also. That’s what I am doing.

Other developmental assignments have been reconstructing the labor room which was not a part of my job description, but still I did that. Then, developing SOPs for the hospital. When the healthcare system came in, the healthcare commission came in, I did their workshops and I helped in implementing most of those things that were told by the healthcare commission into our hospital. I am still a part of that team, the infection control team of the hospital as well as those building up SOPs of the hospital. These developmental assignments have definitely helped build up me. I am not the same person I used to be because I think my qualities have been improved and enhanced and it is only by working on that, in my field, in the practical aspect doing these assignments that have helped me build myself as a better person, a better leader.

**Q: If we dig a level deeper, what was it about these experiences that made them a positive learning experience?**

The main thing was that these tasks were not concerning me, these were concerning a development that would affect many people who wouldn’t even know that I had done something good. I was being guided on international grounds, I had international data behind me. I knew what I was taking up was being done in many places and it had brought out a good outcome. So if I could implement it on my home ground, I thought I would be able to challenge and bring something good for everybody to share.

**Q: Certain characteristics of tasks that help in leadership development have been identified in the corporate sector. Can you please elaborate on and relate to them with respect to clinical tasks?**

Q: High levels of responsibility: Do you think that high level of responsibility in a task can help in developing leadership skills in the person performing the task? Also, how does this occur and can you relate to it in the clinical scenario?

When you are the only one who is being relied on makes you very strong. I have been through that and it tells me that I am somebody who is active, more critical as well as more helpful towards people. For example, when you are teaching a small surgery to a trainee of yours, you are taking the responsibility of the life of a patient into your hands, even though the hands performing the surgery are not yours but your eyes, your mind should always be there. And I am the one who always takes the responsibility. I sit down with my trainees, I help them throughout the surgery until they are in a safe ground.

Q: Creating change: How do you think a task involving creating change of any sort will affect leadership development in the person performing the task?

Yes, definitely. No matter how small the change may be. However, getting acceptance from the other side, from the people whom you are bringing the change on might be difficult. There would be some who would be your supporters but mostly you will find will be against you and then convincing them to bring them to a point where they can rehearse it and once they rehearse it, then you can give them your feedback weighing the pros and cons.

Q: Unfamiliar responsibilities: Do you think that a task involving unfamiliar responsibilities can help in leadership development? Can you relate to this from your training?

Well, first of all, I have to get down to the details of that task because until and unless I am sure about the benefits of a task, I will not take it up. If I am sure that the thing that I am going to do is going to benefit most of the people around me, then I will definitely take it up. However if it is to fulfil some small purpose that is probably going to make somebody’s pocket heavy or just help somebody on a high authority, then I might not be a very suitable person for that.

Q: Working across boundaries: How do you think working across boundaries helps in leadership development in clinical trainees?

I am someone who believes in teamwork. I do not believe in one-man show. I have done a lot of surgeries but I always take the precaution of having a surgeon around. If a surgeon can come and just sit in the next room, it would be a big solace for me knowing that there is somebody who can be called for help> In some instances, I have gotten a lot of help from a surgeon and I have learned from that. I don’t feel shameful in asking for help if I need it or if my trainees need it. I think that one should focus more on teamwork. My field is a very sensitive one and if I feel that I am not trained enough to do something, then someone who is trained enough should do it.

Q: Managing diversity: How do you think a task involving managing diversity can affect leadership development in the clinical trainees performing the task?

I might be teaching the same topic to different level of students like it could be the postgraduates, the undergraduates or the nursing staff and if it is a disease, I could even be teaching my patients about it. So my way of teaching will have to be different. The amount of information may vary. I think I do that here. My patients are very happy because I teach them what is happening and explain to them. It creates a good environment. There have been instances where my students say that we don’t want to study, we want to watch a movie, so we watched a movie regarding the medical profession, the emergency. It was a different mode of communication and since my students were more adaptable to that, I thought it would be better to generate their interest and communicate the knowledge.

**Q: As a clinical leader, you assign tasks to you subordinates/juniors. Do you consider incorporating certain elements/characteristics in those tasks in order to help the assignees grow as leaders?**

Well, definitely. I believe in advancing just like I have taken up a new unit now. They have fixed tasks for everyone in the unit, the residents and the senior registrars. Their level of training depends on that. I believe that a person assigned to a certain label should rotate to all the responsibilities of that label, for example a senior registrar should have training in teaching, administration, skills development. I don’t believe in holding one person to one task, rather everybody should get a taste of every task that they have to perform.

Q: What do you think should the level of autonomy while assigning a task?

The first thing to keep in mind while deciding the level of autonomy is what is at stake, that is, patient safety. You are dealing with a human life. So the safety range has to be considered. You cannot give an assignment to a house officer which is the lowest level of a person on the clinical ground something that is totally dependent on him. They are there to be supervised by the person who is immediately senior to them or any senior. So whatever is chosen to be given to them as an assignment, it has to be something within a safe range and it has to be supervised.

Q: If you feel that a first year resident has the caliber of performing a second or a third year resident’s tasks and you feel that doing so will enhance leadership development in that trainee, will you assign a higher level task to him/her?

The thing is that I have to be a fair leader. If I do that for one person, the other persons will feel that I am probably doing a favor to him. I will have to assign tasks at a general level and then I have to develop confidence at a general level that all are in agreement. If the agreement is there then we come to a point where we cannot limit ourselves to one task, we have to multi-task, for example, that person might be given something like an academic task. That might involve guiding the juniors, taking classes or preparing the lectures. That could be helpful in developing his leadership skills. As far as surgery is concerned, he/she might be given the surgery as a reward or a gift so that can create an environment where everybody will want to perform well and get that gift. That will be my kind of approach.

Q: What, in your view, is the effect of Briefing before assigning a task & Debriefing after the completion of the task on leadership development of the trainee performing the task?

This is something very important because when someone is going to perform a task, they should know the reason of performing that task, then you need to tell them what you want them to do, what are the precautions that they will be taking, what could be the advantages and disadvantages, what could be the consequences towards the patient so that he/she can avoid any complications. Once the task is done then definitely debriefing is important. First of all, taking his answers regarding the challenges and then giving him feedback regarding what he/she has done and how it can be improved can be very developmental. Discouragement should not be there. Every task should be encouraged so that performance can be improved.

**Developmental assignments shaping experience-driven acquisition of leadership competencies in clinicians.**

**Interview from R6**

**Duration: 60 min**

**Q. What do you understand by leadership?**

Leader is somebody who leads a group of people or community. In our cases, a leader can be departmental or institutional. I would say that somebody who after defining certain goals and objectives of a given institution and department, decides how to go about them in a collective and responsible way and how to achieve them in a certain framework or a timeline.

**Q. What in your view are the most important leadership attributes that you rely on at your workplace?**

I would say that for clinical leadership, you have to be competent. Leadership can only be done if you are the most competent one in your team. This might not hold true for other managerial jobs but specifically in surgery, you cannot lead if you are not a good surgeon. You can be a relatively average surgeon but then you have to gain the confidence of everybody that you are the one who can drive them forward. If you are competent, most of the times people will follow you. Competency means everything actually. It is not just operating skills. It is the clinical acumen, the behavior or attitude in ward rounds, surgical and organizational skills. These have to be a little above the others or at least one of the best to command respect from the team.

**Q: Literature says that 70% of leadership is developed by developmental assignments (on-the-job tasks). What developmental assignments can you think of, that you would credit, from which you acquired the aforementioned leadership qualities?**

I would give the credit to my supervisor. My supervisor made me go through everything on his behalf. For example, there would be a meeting with administration, he would send me. There would be tasking. For example, ATLS was starting for the first time in Pakistan. He sent me to the first course. In the same go, they wanted instructors. We were selected in the same course and I was made an instructor after two days. Then subsequently in every ATLS and whatever happened with CPSP, I was always involved. In conferences and workshops, he would make me go forward. Because I was the first trainee of thoracic surgery anyway and then subsequently every time I am a fortunate one. My attitude is one of a willing worker. So they would pick me up and task me and when they tasked me, I got to know about things. I am pretty sure that even in people of my seniority, nobody else has been tasked that much the way I have been. It groomed me.

I kept on improving my qualifications. I have done my single FCPS in General Surgery, FRCS in general surgery, thoracic surgery FCPS, then I went on job training, army selected me, then I did a diploma in medical education. My training never stopped and that’s how I never stopped learning. It has been a continuous process.

Personally, I think I have been blessed with a personality and that personality has always been accommodating. Once I came, this department there were perceptions about this department that it would be difficult to get through this department. Right from day one, it has always happened with me that I was the binding force in this department. Each and every trainee who has passed from here have passed in front of me. There might be difference in personalities among different trainees and some of them might not get along so well but I have always had the ability to accommodate them all together. So I think from the very start I was accommodative enough. If you ask me about tasking, I was the senior most, so I took it upon me that there is a problem in the department and I can act as a bridge between the head of the department and the rest of the team. My supervisor was also very good. So we worked more as a family than a true department in its medicals sense.

**Q: If we dig a level deeper, what was it about these experiences that made them a positive learning experience?**

High level of responsibility was always there. Second is creating change. Yes, there were unfamiliar responsibilities and creating change. This department has grown from infancy to a stage in which it has grown to one of the best departments at least in Pakistan as far as diversity is concerned, maybe not the numbers. We started from scratch actually, in the sense that there were very few and limited procedures that were started. Each of the procedures that were started to add to the diversity, I was personally responsible for it. So creating change was always done by me. For some of it, we didn’t even get any formal training. But by studying and listening and video conferencing. Creating change has been very prominent and that has always kept me at the forefront actually. That was one of the aspects through which I think I commanded respect. Working across boundaries and managing diversity were also there. As I told you, I have been multitasking so much that I was able to grow as a leader.

Level of autonomy: I was always autonomous.

Level of autonomy will depend on his level of training. For example, it is his first year. In that way, I think I am a little deficient that I don’t give tasks as liberally as I was given. Because I think that in a structured training, there is a certain level that a training can do. We. On the other hand, were given a very free hand and there were problems in that. So my giving tasks is a little delayed than what I got. It also depends upon whom I am giving the task. The task is gauged upon what the other person can do. Each of my team has certain qualities in which they excel compared to other team members. I task them according to their capabilities. As far as operative work is considered, it is time-bound and I give them when I am sure that they are ready for it because it is then unfair to the patient.

Level of feedback: Feedback is a primary requirement but as far as I am concerned, my feedback is not as structured as it should be probably because we are over committed. I realize that we need to start and sit together and have a proper feedback. In one sense this department is a very close knit department. There are four consultants and five trainees. Feedback is going out in an informal way throughout the day about each other and the patients’ progress but it is not as formal as medical education and as you would like to have it. It is not structured.

There is a certain set of competencies that is required. Feedback should always be about the knowledge, skills and attitude actually. But as far as clinical competence in surgery is concerned, they are well-defined for each year of residency. As far as attitude is concerned, I have more emphasis on attitude, verbal emphasis, not a regular feedback. I personally feel that surgery is difficult but not more than attitude building and it is even more difficult for someone who has been a trained surgeon for a certain period of time and he comes back with a certain background and to make him realize that this is another ball game.

**Q: As a clinical leader, you assign tasks to you subordinates/juniors. Do you consider incorporating certain elements/characteristics in those tasks in order to help the assignees grow as leaders?**

Not specifically. I think it will be more of mentoring that we do here in our department. It is not as such structured leadership skill development. It is participative in some sense and role-modelling.

**Developmental assignments shaping experience-driven acquisition of leadership competencies in clinicians.**

**Interview from R7**

**Q: What do you understand by leadership?**

There are conventional and traditional definitions of leadership. What I understand by leadership, particularly in the clinical perspective is the liberty of thought, the ability to make decisions uninfluenced by any extrinsic effects, exercising of authorities with justification and accomplishment of delegated tasks using independent thought and independent decision making. It also includes delegating the tasks to other people, working with teams, understanding the capabilities and otherwise of individuals and deciding about delegation of work according to the abilities of people. It also includes scaffolding the juniors and looking closely and supervising closely the work of juniors and taking appropriate actions as and when required.

**Q: What, in your opinion, are the leadership qualities in you that you rely on at your workplace?**

I think understanding the individual capabilities and shortcomings is one of the most important leadership qualities that are utilized while you are behaving as a leader, making a team, trying to join people to achieve common and shared goals and giving them different assignments or delegating them different tasks, giving them time targets to achieve those. Another leadership quality that we have to rely on at our workplace is the amount of motivation that you impart to your juniors and this has got close relationship with our actions and decisions and verbal commands given to the juniors and members of the team. Another important element in clinical leadership is the amount of independence and liberty of decision making particularly clinical decision making related to patients. Have to take into account individual capabilities but at the same time should ensure that no aspect of patient is compromised.

**Q: Literature says that 70% of leadership is developed by developmental assignments (on-the-job tasks). What developmental assignments can you think of, that you would credit, from which you acquired the aforementioned leadership qualities?**

As I can recall, most of these tasks were related to our clinical work which was related to patient care, surgical decision making and accomplishment of surgical procedures. I as a resident was supposed to perform emergency operations and make sure that no emergency is unduly delayed or postponed for the next day’s operation list. So we were actually free to decide about surgical procedures after receiving patients in emergency and accomplish those decisions in the form of surgical procedures, do them independently or take the help of senior residents or senior surgeons in order to accomplish those surgical procedures. This was an activity that served to promote independent surgical decision making, assuming responsibility, taking care of patients, counselling of the attendants, making social relationships with social awareness of the patients’ predicaments, the predicaments of their attendants and taking into account all the picture frame factors which underlie a surgical procedure and operative intervention and morbidity and mortality of the patient according to their surgical condition which pose a challenge to the clinician. The other activity that probably contributed towards development of leadership qualities was managing the undergraduate medical students and working with the house officers, development of a team, sharing simple tasks, delegating tasks to the team of house officers and accomplishing different tasks with the help of house officers and other staff like nursing staff and the rest of the management. This helped promote the ability to work with teams, collaborate, delegate tasks, assign tasks to different people, meeting task targets and maintaining quality in our work in order to avoid strict accountability that would usually entail these procedures. The next type of activity was related to our clinical teaching. As residents, we were supposed to reach our juniors, undergraduate medical students and junior residents and that teaching would involve quite a lot of leadership or leadership like activities that would entail effective clinical teaching. This was also a help for our own development as clinical teachers as well as was a requirement by the degree awarding institutions so that we had to take part in teaching activities. Preparing different clinical cases for the purpose of presentation and publication and developing different types of research protocols was a minor part of our activities that contributed to development of leadership qualities. The managerial tasks mostly consist of administering the house officers as senior residents. We would receive a large number of house officers, about 10 to 20 in each session, making teams, making rosters, rotating them in different specialties and different areas of work like opd, ward, operation theatre and emergency. That was a managerial activity that contributed towards leadership development.

**Q: If we dig a level deeper, what was it about these experiences that made them a positive learning experience?**

I think the main characteristic was the liberty of making independent decisions. Of course it was associated with a certain amount of accountability as well but on this part we were independent to decide what we are supposed to do and how we are supposed to behave. The next thing was the nature of different tasks, mostly related to operative procedures, but sometimes related to organization of academic activities, timetabling and management of other junior trainees, coaching the junior medical students, undergraduate and postgraduate students, that would make these a positive learning experience and that would also promote the qualities of leadership. Working in collaboration with lower and higher administration from time to time would also become a subject of discussion as well as changes in the actions and decisions would take place because of these and that also would be a positive learning experience. Accidental events, particularly I remember the 1988 Ojhri Camp blast that gave us an opportunity of disaster management as an accident and all the activities related to the disaster management: coordinating the work, receiving the patients, developing different teams at different levels and on the spot decision making was something that would contribute greatly as a positive learning experience for promotion of leadership qualities.

**Q: Certain characteristics of tasks that help in leadership development have been identified in the corporate sector. Can you please elaborate on and relate to them with respect to clinical tasks?**

Q: High levels of responsibility: Do you think that high level of responsibility in a task can help in developing leadership skills in the person performing the task? Also, how does this occur and can you relate to it in the clinical scenario?

Q: Creating change: How do you think a task involving creating change of any sort will affect leadership development in the person performing the task?

I think the most major change that I have encountered in my professional life is the change in our curriculum. With the advent of new thoughts in medical curricula, development of integrated curriculum was one major change that was well-managed and well taken care of in my institution in a span of about 5, 6 years over which this change has occurred. Starting from basic academic level, changes in curricula document and going up to the level of faculty development and managing the faculty resistance as well as promoting faculty developmental activities related to this major change.

Q: Working across boundaries: How do you think working across boundaries helps in leadership development in clinical trainees?

Working across boundaries is a tricky terminology. Working across boundaries may on one hand mean encroaching upon others’ boundaries which is probably not the right way of describing working across boundaries. Another meaning that can be assigned to this term can be collaborating across boundaries with people from other disciplines, people of other specialties and I think that is something that positively contributes towards leadership development. But encroaching the boundaries of others and crossing one’s own boundaries without any collaboration with other people is something which is not regarded as developmental.

Q: Managing diversity: How do you think a task involving managing diversity can affect leadership development in the clinical trainees performing the task?

Managing diversity is our day to day job. Maintaining interdisciplinary coordination and maintain a multidisciplinary environment for patient care is our day to day job and in my experience it helps in leadership qualities. However I don’t think it requires designing any deliberate activities. If managing diversity means working in diverse professional environments, this is something that is hardly ever possible in our environment because most of the times we work in a fixed environment for a long time. It is related more to the field workers, people who work in different teams, who work in the periphery or sometimes related to training different people at different levels.

**Q: As a clinical leader, you assign tasks to you subordinates/juniors. Do you consider incorporating certain elements/characteristics in those tasks in order to help the assignees grow as leaders?**

I think whenever we assign, rather delegate a task to some person, we should have profound awareness of the abilities and shortcomings of that individual. The task should be delegated in such a manner that the short comings are properly accounted for. Second thing that should be a part of this is that whenever a task is delegated, it should entail necessary authorities as well as necessary resources to accomplish that task. Assigning time targets which are logical to accomplish a task is also something that would promote the meeting of time targets as well as quality targets. The time target and quality target of the delegated task should be clearly pronounced and should be clearly understood by the person who is given the task and then if any targets, quality or time, are not met properly at any stage of the task accomplishment, some training or learning activity must entail that activity so that the shortcomings are overcome. Problems related to the inability to meet the targets should be recognized and properly taken care of.

Q: What, in your view, is the effect of Briefing before assigning a task & Debriefing after the completion of the task on leadership development of the trainee performing the task?

They are essential. Without briefing, a task cannot be properly delegated and that must include the time targets, the quality targets as well as the necessary information in order to overcome the shortcomings of the individual who is being delegated the task and obviously if the time target has been announced, a debriefing has to take place when the time lapses.

**Developmental assignments shaping experience-driven acquisition of leadership competencies in clinicians.**

**Interview from R8**

**Q: What do you understand by leadership?**

**Q: What, in your opinion, are the leadership qualities in you that you rely on at your workplace?**

There are ten leadership qualities that every person who is a leader must have: the first thing is integrity. If I am a leader and I do not have integrity, my juniors are not going to follow me. I have to be honest and punctual and make sure that everything that I do is according to ethical values. The second value that I should have is honesty and be able to inspire confidence in my followers. If I am not honest and everything that I do is dishonest and unethical, then thing do not work out so well. The third thing is confidence. If I am a leader and I am confident, then I can lead others also. If I am not sure about whatever I decide, for example, if there is patient with placenta percreta which is a life-threatening condition, my one decision can be death for the patient. If I am not confident, my juniors will say that she makes wrong decisions and does not have any leadership qualities. So I have to be confident to instill confidence in others. The next one is I must inspire others and I can do that only by setting a good example, if I do everything correctly, my leadership role is good and I do justice to everybody. If I do favoritism with my trainees, nobody is going to consider me as their leader. Then is commitment and passion. I am the head of my department and I am not passionate about my work, I do not come to work every day, I am not interested, I do surgeries but half-heartedly, I do ward rounds but in a disconnected way. If I have passion and I want to do things rightly and correctly, only then I can be a good leader and I have to show that and instill that into my juniors. I have 0 doctors working under me. If I don’t do that, I don’t look committed and do not inspire passion, then the juniors are also not going to work properly. I have to be a good communicator also. If I want something done in my department but I didn’t communicate well along the hierarchy, they will not be able to do the task well. So I have to follow my hierarchy, I have to communicate well and to do that, I have to take people into confidence, clearly communicate my commands, not just tell my assistant to tell everyone and not directly talk to everyone. Until and unless I give clear cut commands and communicate well, I cannot be a good leader. If there are four patients on the list and I want to operate the first one myself, I want the second and third ones to be operated by the associate and assistant professor respectively, I have to communicate this to the right person in the perfect tone so that the person knows what is being communicated to him/her. Decision making is a very important part of being a leader. Accountability is also very important. As a leader, I am also accountable. I should not be above accountability plus my juniors should know that they are going to be accountable for whatever they do. If they know that this accountability runs all the way from top to bottom, then they will consider me their leader. Delegation and empowerment also plays a major role. I cannot do everything alone. It has to be teamwork. Teamwork is an integral part of leadership. If I am not leading a team and I want to do everything myself, I want to do the opd myself, I want to do all the surgeries myself, I want to teach the students myself, I want to make the five-year plan for my department myself, I want to start any new project myself, then it will not be possible at all. I must empower my juniors. Some tasks I will allocate to my associate, some to my assistant and some to my trainees. For example, I have empowered my assistant and associate professors to conduct the fourth year OSCE themselves. Then there is creativity and innovation. If you remain stagnant, you lag behind from the rest of the world. If all the world is doing hysteroscopies and has abandoned DnCs and I am still doing DnCs, then I am not a good leader. I might have to convince and push my seniors and management for the change and provide finances but I have to bring the change in my department for my juniors. If all the hospitals in Pakistan are taking part in an international conference and I do not take part in it, then I will be left behind and I will fail my trainees. Out of all these leadership qualities, I think integrity is the most important.

**Q: Literature says that 70% of leadership is developed by developmental assignments (on-the-job tasks). What developmental assignments can you think of, that you would credit, from which you acquired the aforementioned leadership qualities?**

I have always been involved in undergraduate teaching. When I was a PG trainee, my supervisor would let me take me classes of other PG trainees and undergrads. I think that helped me developed my confidence. Also, I had to take their exams. I used to take long cases of undergraduate students. That instilled confidence in me. I was involved in research by my professor and supervisor. They told me to write papers and do research. They helped me develop my leadership qualities. When I was a PG trainee, my supervisor would just sit in her office and tell me to do the whole operating list. She helped me just once or twice, for example, she did one abdominal hysterectomy, one vaginal hysterectomy and one laparoscopy and she told me to assist me. The next one she said I am sitting in my office, call me if you get stuck. I knew she was nearby and I could call her whenever I needed her and that instilled great confidence into me. That helped me develop my leadership qualities. We had an MBBS exam and I was told by my supervisor that you have to arrange the exam and you have to arrange the tea and lunch for the examiners, I had to do all this for eight days straight. Which examiner will come at what time, who will take how many students, making list of students, dividing them into senior four examiners. These managerial tasks helped me in developing leadership skills.

**Q: If we dig a level deeper, what was it about these experiences that made them a positive learning experience?**

I think the confidence that was instilled in me to lead the team, that helped me develop my teamwork, that helped me develop in my responsibilities and developed ethics and integrity in me because I had to do everything honestly and in the correct way.

**Q: Certain characteristics of tasks that help in leadership development have been identified in the corporate sector. Can you please elaborate on and relate to them with respect to clinical tasks?**

Q: High levels of responsibility: Do you think that high level of responsibility in a task can help in developing leadership skills in the person performing the task? Also, how does this occur and can you relate to it in the clinical scenario?

We had an international conference in obstetrics and gynecology and my supervisor thought that it was a very high level of responsibility. I had to plan out and divide my time, make a time table and then put the plans into practice. Then the event happened and it went very well so I think it helped me as it was a very high level of confidence as the outcome was well.

Q: Creating change: How do you think a task involving creating change of any sort will affect leadership development in the person performing the task?

We were initially into conventional method of teaching. We had to switch over to problem-based learning and CBLs and modular system. I think I did that well. I shifted from conventional to the modular system. It brought a lot of change in me because of the creativity, my mind worked well. I think I improved myself as a leader in doing that. That made me more confident.

Q: Unfamiliar responsibilities: Do you think that a task involving unfamiliar responsibilities can help in leadership development? Can you relate to this from your training?

We had ISO certification and the first ever patient safety conference in Pakistan was conducted by Riphah University. I had a leading role in that. We had to do the patient safety conference. Although we practice patient safety all pour lives but to convert it into the format of a conference, it was completely unfamiliar to me and that was a great experience. Also for ISO certification, even though we were doing everything as doctors, but to streamline it, divide it into portions and exactly doing according to the checklist, that was a new thing that I experienced.

Q: Working across boundaries: How do you think working across boundaries helps in leadership development in clinical trainees?

Where I was doing my PG training, it was a just a Paeds and Gynae hospital and the other hospital where the surgery and the medicine people were available was at a distance, so that’s how I worked out of my boundaries. I had to repair the bladder so many times because the urologist was not available. I had to repair the gut also. That helped leadership in me.

Q: Managing diversity: How do you think a task involving managing diversity can affect leadership development in the clinical trainees performing the task?

We have an LHV program. It is a one year program and I am in-charge of that program. I have to teach them and I have to find out the curriculum, which curriculum is going to fit into that. Initially when we started it was a bit difficult. Now this program has been going on for many years, so I am helping the nursing head with that also. The first thing I do is how to select these girls for the LHV program. So I have made a criteria to select them. So this is apart from my duties as a doctor and has helped me develop my leadership skills.

**Q: As a clinical leader, you assign tasks to you subordinates/juniors. Do you consider incorporating certain elements/characteristics in those tasks in order to help the assignees grow as leaders?**

I give my trainees the guidelines that you have to do it like this. For example, we are going to start a Masters program in Obs and Gynae, so to do that, I have to first find things out myself. I have to do the research and find out where such programs are being run, how successful are they, what is the outcome and the outline I had to work out myself. Then I asked two of my juniors to help me out. I gave them the outline and I told them to develop the program themselves. So I am helping them become leaders also. They are my future. Another example that I can give you is that there was a time about five years ago when three of my very senior doctors suddenly resigned and left but no vacuum was created and the reason for that was because my junior team was ready to take over. It went very smoothly. No chaos was created and no problem occurred.

Q: What, in your view, is the effect of Briefing before assigning a task & Debriefing after the completion of the task on leadership development of the trainee performing the task?

We must tell the juniors what do I expect, give them the task and let them do it and after they are done, they will bring it back to me. Then I go through it and find out if there is a problem with that and then I can debrief them. If it is good, I always help them, congratulate them, and boost their confidence. It actually has to be PNP, that is positive, negative, positive. I first appreciate what they have done. If there is some problem in the task that they have done, I tell them that they have to correct it and in the end, enclose it again that this is a great job that they have done, only correct these problems and you are good to go.

Q: What do you think should the level of autonomy while assigning a task?

Q: If you feel that a first year resident has the caliber of performing a second or a third year resident’s tasks and you feel that doing so will enhance leadership development in that trainee, will you assign a higher level task to him/her?

No, I think we should stay in the confined areas which have been allocated. Autonomy as such would create a bifurcation in the division of the PGs. The issue of fairness might arise. So I would suggest that it would be better if every person stays in their own domains. If I am fair to one person, then I have to be fair to the others also.

**Developmental assignments shaping experience-driven acquisition of leadership competencies in clinicians.**

**Interview from R9**

**Q: What do you understand by leadership?**

Leader has a vision or a way of doing things the way he perceives things and he inspires others to actually follow that. This inspiration actually comes from the way he has a relationship with the people who are down the line or who are in his followership. Clinical leadership involves getting the trainees to follow you as a role model. They observe you that this is the way things have to be done, they should be done, this is the path that they can take and the leader helps in making the path shorter. A leader makes the followers’ task easy for them instead of making it more difficult. There is a limited time period in which clinical trainees have to learn and there is a lot to be done and learnt during that time, so empower them and show them the direction to make the task easier and more understandable for them.

**Q: What, in your opinion, are the leadership qualities in you that you rely on at your workplace?**

I think whenever there is a task at hand, it is important to understand the task and be able to delegate it accordingly. I think a leader cannot do everything him/herself, so I empower my trainees. When you are leading a department, there are certain things that can be you as well as others, so we need to empower. There are times when you need to understand and identify what it is that they can do, what is something in which they need to have my supervision. They should know when to call me. I don’t take everything all the time. I just have to stand there and bear the brunt of teaching and waiting. So I think identifying, empowering and patience are my key leadership qualities. I usually wait it out while training. There are multiple tasks in histopathology. Microscopy is done under constant supervision, but when there is a gross cut up, I have to leave the trainees alone, even with my technicians and even with my students. I train them and when I train them, I identify what are the tasks that they can do entirely on their own, what are the tasks where they must call me for supervision, so I have to set the boundaries.

**Q: Literature says that 70% of leadership is developed by developmental assignments (on-the-job tasks). What developmental assignments can you think of, that you would credit, from which you acquired the aforementioned leadership qualities?**

It was a four year training period for me and what we are doing now is a two year training period because we mostly get MPhil trainees. I think a lot of things helped me in developing my leadership qualities. Empowerment was a big part of my training. I had my own batch for which I was totally responsible, its timing, its grossing, everything. So when you have responsibility and ownership for a task, then I think leadership develops. Ownership leads to building own leadership because you have to do something entirely on your own. Also, we had exercises where we used to get a topic or some reading material, like we do in MHPE. We had to do our own research on it, then we had to go and teach as well as present on which a critical analysis will be done. So all these tasks, teaching tasks, presentation tasks as well as tasks involving critical thinking as well as empowerment, these were the factors contributing towards leadership when you are finally in a leadership position and you have to take decisions. If you have not gone through this all, then decision taking, teaching, presenting in front of a large group becomes difficult. I practice pathology. This involves a few things: tissue pathology, communication with patients where we have to draw the cytology samples from the patients ourselves. This task in entirely in the hand of trainees. They have their own clinic where they do the FNACs themselves. The supervisor teaches them on day 1 and supervises a couple of times, then leaves them alone. They might have to repeat them in the beginning a couple of times. You have to teach them how to communicate, this is how to do the task adequately and then rely on them and then you cannot leave them unsupervised for microscopy. I have to impart these communication skills related to patient encounters the way they were imparted to us by our seniors during our training. The cases that we receive are basically tissues. We have to do grossing of these tissues and that is a totally empowered job. If we need any added information, how to contact the respective department, the patient, the doctor, all this has to be done by the trainee. The microscopy part is the one that is always done under supervision and then we have to teach them that microscopy depends upon what you bring, that is, grossing and communication. Picture matching comes later. We have to teach and guide them differentials, guide them what to see and what not to see and how to go about things in a structured manner. Also, how to manage your time well, because there are days where you are doing different tasks. A trainee gets different cytology batches and he/she has to show them to his/her supervisor. So he/she has to have something on the plate to give to the supervisor. Initially people are not able to manage time but there is a timeline that has to be followed. Managerial tasks related to my job are basically academic in nature. The trainees have to manage undergraduate teaching themselves. In our program, the focus of trainees is mostly on undergraduate teaching and research and then clinical work. Since histopathology is related to clinical work, so we focus a lot on work place but undergraduate teaching and lab management are also important. Management of the department is also something that our trainees have to do. There are four or five subfields in pathology and the trainees have to get exposure in all of them, even if they are training in just one department. So the trainees have to manage their clinical work, their academics, their teaching along with their lab work and lab staff. Management also involves management of the situation and coworkers for example, who will be present here and who will do this task in the morning, the work should not stop. We ask them and teach them to manage such things and communicate among themselves. In my postgraduate training, patient dealing is the sole responsibility of the trainee. So management of communication with the patient at all levels, timing and inter-departmental communication was our responsibility during training. Also there used to be CPCs and one trainee was always delegated the task to manage them.

**Q: If we dig a level deeper, what was it about these experiences that made them a positive learning experience?**

Q: High levels of responsibility: Do you think that high level of responsibility in a task can help in developing leadership skills in the person performing the task? Also, how does this occur and can you relate to it in the clinical scenario?

If there are four years to your training and you are in the final year of your training, so to speak, on the hot seat and are empowered to a degree where your supervisor can trust you to some degree and so to speak, the junior trainees also come under your supervision. This is also a level of empowerment that you will train the junior trainees to some level for the stuff that you can do entirely on your own. Then there are always certain cases that require a lot of studying and background knowledge that also needs to be taught that there would also be certain cases where consultation would be needed, how to save your skin and how to ask for external output or an additional test from someone. A trainee has to recognize his/her own limitations and know when to ask for consultation to ensure patient safety.

Q: If you feel that a first year resident has the caliber of performing a second or a third year resident’s tasks and you feel that doing so will enhance leadership development in that trainee, will you assign a higher level task to him/her?

I do stretch them cautiously and empower them as I feel that it is a wastage of time if you do not do that. If they are able to do higher level tasks, I give them those tasks without compromising patient safety. The issue of fairness does not arise as it is a one on one communication. The report is written by the resident but I always second it, so I never let go of that report without it having gone through my eyes. When you have trained a resident from the very beginning, you know where a problem can arise so you do catch the problem. Then there is a 360 degree evaluation. You have your peers, you have your technicians.

Q: Creating change: How do you think a task involving creating change of any sort will affect leadership development in the person performing the task?

I don’t remember in my experience while I was being taught but we actually plan a change for here. There are a lot of things going on according to bookish knowledge and if you want a change in that, you have to plan accordingly. You have plenty of time for a four year training program but we had to design a two year program, so we had to plan for it that what tasks will be accomplished in what way, what will be done and taught at the workplace, what should be the level of the assignments, so you need to actually bring out such assignments. If we need change, we need to plan for it and communicate it to the trainees. That does help in leadership development, because the trainees develop self-control and self-tolerance. They also develop communication skills, they learn how to communicate with their peers, their subordinates and their superiors. This comes under the umbrella of professionalism as well as leadership. They also learn how to talk to people when they need information, how not to talk to people.

Q: Unfamiliar responsibilities: Do you think that a task involving unfamiliar responsibilities can help in leadership development? Can you relate to this from your training?

Everything that we learned, we did it as an unfamiliar responsibility and everything we give our trainees, the poor things is always unfamiliar. When we give our trainees lectures, we just tell them that it’s a problem-based approach that you have to use. Everything we tell them to do in the beginning, its Greek to them. We have to train them how to go about it. When you are in your comfort zone, you can do the task in its prime but there is always a first time for something, when you stretch yourself, you learn and it is only possible by giving unfamiliar responsibilities. Such tasks widen the trainee’s horizon in a way that books cannot.

Q: Working across boundaries: How do you think working across boundaries helps in leadership development in clinical trainees?

The boundary between postgrad and undergrad is not very distinct in pathology so we definitely have to work across boundaries. The undergraduate teaching involves a lot of working across boundaries because we have to tele with different departments. In clinical setup in our hospitals, there is still not a well-developed system of formal communication. Informal communication is there though. Sometimes we do need to develop informal relationships at personal level. Inculcation at a formal level is lacking and steps should be taken to develop it. Medical education also plays a role in this regard, bridging the boundaries and making it easier to communicate. You get to know a lot of people.

Q: Managing diversity: How do you think a task involving managing diversity can affect leadership development in the clinical trainees performing the task?

There is diversity in students as well, so managing their problems, having psychological knowledge etc is important. When you are a supervisor, you get difficult trainees. They have various backgrounds; educational, psychological, which are a part of them. You have to take them accordingly based on their level and their psychological buildup. You have to identify people and train them accordingly. It varies from trainee to trainee, who you have to talk to firmly and who you have to talk to leniently. That should help in leadership development.

The only diversity that we as histopathology trainees encountered is the many types of patients that we have to manage and many departments that we have to communicate with. There is diversity even at the level of academics, for example, general histopathology will mean skin, neuro, kidney, liver, everything and accordingly all the various departments. The needs, requirements and time frame of every department if different from the others. We had to deal with multiple types of departments. The way you approach a case from skin or breast is completely different. If for example, pleomorphism is a sign of an abnormality in one tissue, the lack of pleomorphism is an alarming sign in another tissue. So one thing is a positive sign in one tissue whereas the same thing is a negative sign in another tissue. Department dealings vary according to their requirements and time frame. Skin reporting will be entirely different from a surgery or a neurosurgery report. The information you get from them will be different.

**Q: As a clinical leader, you assign tasks to you subordinates/juniors. Do you consider incorporating certain elements/characteristics in those tasks in order to help the assignees grow as leaders?**

What I do is that when they initially come, there is an orientation period and we have to teach them everything (haath pakar ker sikhana) in that period. Orientation period isn’t more than 2-3 weeks. We empower them along the way and we encourage them to start writing whatever they see because when you write, you make a commitment. We teach them the approach of commitment so that they can lean the differentials. We do not snub them initially because it takes time. When there is a commitment, them they have that thing in mind and they can work around it. If they do not commit, then there is an ambiguity and they cannot give arguments for and against their point. So I think commitment is important and they have to bring it in a written form. For future leaders, it is important to commit, to communicate with others, to focus on something, to bring yourself towards something, you need to develop a thought process. When you are committing, you need to communicate what are the points you have that actually help towards those, why did you commit to that. Secondly, there is confidence building. Third, when they commit, they feel empowered as well. If we don’t teach them to commit since the beginning, it changes at the point and they are not able to develop that confidence.

**Developmental assignments shaping experience-driven acquisition of leadership competencies in clinicians.**

**Interview from R10**

**Q: What do you understand by leadership?**

In my opinion, a person who has got some vision and who is leading a group of people and who helps them to set different goals and motivates and guides them and helps them to follow him to achieve those goals is a leader.

**Q: What, in your opinion, are the leadership qualities in you that you rely on at your workplace?**

I think the most important thing is honesty and I can alhamdulillah claim that I am honest. I am clear in my mind what I want my followers to do. This is important because until and unless a leader’s concepts are clear, he will not be able to motivate the others to follow him to achieve their goals. I am hard working. I don’t have an authoritative style. I call all my subordinates and I discuss the issue or task at hand with them and get their view point. Then I make the decision in the light of their view point. I take them on board. I give them the feeling that they were involved in the decision making. So collaboration is my approach. Then I have a strong concept of reward and punishment. If someone does anything good or if my department gets any credit from higher authorities due to any task, then I buck up my employees a hundred percent. If my subordinates get caught due to some mistake that they have made, then I protect them. I face the consequences themselves. My bosses sometimes tell me that I am being over protective, but that is my style. I try to keep my employees accountable myself and punish them on any mistake myself but I try to protect them from higher authorities and management. I communicate well with my employees and repeatedly discuss the matters with them. I give a lot of motivational talks to my employees because I feel that extrinsic motivating factors are more important for any person as compared to intrinsic motivation. My concept is that your students or trainees learn what you show them, not what you tell them. So I try to become a role model. I try to motivate them and get them on board with my actions and my efforts instead of imposing on them. I think I am a bit of a hard master. I punish the person who makes a mistake and I think it is important to do so. I feel that I am flexible. It is not that I am not confident in making decisions but I am not over confident. At times my trainees say to me that this thing should be like this, I listen to them and give weightage to their opinion and I sometimes change my decision based on their opinion.

**Q: Literature says that 70% of leadership is developed by developmental assignments (on-the-job tasks). What developmental assignments can you think of, that you would credit, from which you acquired the aforementioned leadership qualities?**

When I was in Armed Forces Institute of Pathology, my boss used to give us a problem, for example, if a patient’s or chief’s wife’s report went missing, he would ask us to handle the problem ourselves. He would give us a free hand. He asked me to go and deal with the situation and get back to him. I used to solve such problems resulting in confidence building. Secondly, he was very mild and understood our problems. I also think that leadership and personality development also depend on many other people and factors, even your children. Sometimes, you learn even from your patients. We attended a lot of motivational talks also but I learned the most from my role models.

When we were doing FCPS training, we were asked to search ourselves and come up with a topic and then discuss with the seniors. We were left in the open sea. They did guide us but they did not spoon feed us. I feel that led to my growth academically and as a leader. When I came here, my boss asked me to get the department recognized by PMDC and HEC. He did not give me a guideline on how to do it. I found out that what you learn with your hard work, effort and involvement always stays with you and it is always long lasting.

**Q: If we dig a level deeper, what was it about these experiences that made them a positive learning experience?**

Giving a free hand is important in giving them confidence and motivating the trainees. Communication is also important, also give them inspirational talks.

**Q: Certain characteristics of tasks that help in leadership development have been identified in the corporate sector. Can you please elaborate on and relate to them with respect to clinical tasks?**

Q: High levels of responsibility: Do you think that high level of responsibility in a task can help in developing leadership skills in the person performing the task? Also, how does this occur and can you relate to it in the clinical scenario?

Such institutional problems were given to me with high levels of responsibility that helped me in developing leadership skills.

Q: Creating change: How do you think a task involving creating change of any sort will affect leadership development in the person performing the task?

A justified change did not occur in my training. There was a lot of talk and discussion about bringing change but there were always limitations regarding either resources or infrastructure that blocked the way.

Q: Working across boundaries: How do you think working across boundaries helps in leadership development in clinical trainees?

Working across boundaries helps in personal growth. Here I am HOD pathology and I was given a task as the managing editor of a journal. This was not in my job description. I was also made a member of the scientific committee for the ICME. I was asked to check all the received abstracts, filter them out and also compile an abstract book. These duties helped in my personal development and contribution towards my institution.

Q: Managing diversity: How do you think a task involving managing diversity can affect leadership development in the clinical trainees performing the task?

Managing diversity gives one inside satisfaction. If you are working with people and you do not connect with them. I try to connect with my students, trainees and technicians.

**Q: As a clinical leader, you assign tasks to you subordinates/juniors. Do you consider incorporating certain elements/characteristics in those tasks in order to help the assignees grow as leaders?**

I give my trainees a free hand while giving them a task. I give them a goal. That goal is set by collaborating with them and motivating them. Then they are given the liberty to move according to their own way. Once they have performed the task and performing it in their own way has resulted in a good outcome, I always encourage them to increase their confidence.

Q: What do you think should the level of autonomy while assigning a task?

I am in favor of controlled autonomy.

Q: If you feel that a first year resident has the caliber of performing a second or a third year resident’s tasks and you feel that doing so will enhance leadership development in that trainee, will you assign a higher level task to him/her?

I assign competent trainees tasks of a higher level. That will give him more confidence and will be a source of motivation for other trainees.

Q: What, in your view, is the effect of Briefing before assigning a task & Debriefing after the completion of the task on leadership development of the trainee performing the task?

Briefing and debriefing are very important because the trainee will not be able to follow you unless you properly communicate your vision to him/her.

**Developmental assignments shaping experience-driven acquisition of leadership competencies in clinicians.**

**Interview from R11**

**Q: What do you understand by leadership?**

Clinical leadership involves whenever you are dealing with people; your colleagues as well as the patients. These are the main stakeholders. Clinical leadership involves effectively controlling, facilitating and teaching them in an efficient and more effective way.

**Q: What, in your opinion, are the leadership qualities in you that you rely on at your workplace?**

First thing is hard work, second thing is competence. Competence means that you are conversant with your profession and you must have the knowledge, skills and attitude related to the particular subject. Third thing is the communication skills and fourth is administrative qualities. But over and above all this is hard work. If you can do hard work and are honest, then you have the four pillars of leadership. This is supplemented by three Ds, that is, dedication, devotion and determination towards that task.

I think I have four qualities in me that I have based my life on. First thing which is the main key is the hard work. The second thing is my hold on the subject, third thing is dedication and fourth is honesty.

**Q: Literature says that 70% of leadership is developed by developmental assignments (on-the-job tasks). What developmental assignments can you think of, that you would credit, from which you acquired the aforementioned leadership qualities?**

When I was working at Lady Wellingdon Hospital, my mentor and role model was DR. Yasmeen Rashid. She used to send me to television and live radio programs and also gave me opportunities to teach students. At that time, it was very challenging for me to speak on the radio while it was being broadcasted live, but created a lot of confidence in my personality. Similarly when I was teaching the students in an extempore manner, without any preparation or notes, which enhanced and enabled me to work on my capabilities, my education, as well as my knowledge. At that time, I was not a medical educationist or a teacher. I was just a post graduate resident. So that really polished my abilities to talk in front of students and that is why that enabled me to become a good leader.

When I was working as a postgraduate trainee, I was given independent duties to perform cesarean sections. Someone would be backing me but not at that place, somewhere else, backing me and encouraging me to do cesarean sections and handle emergencies. So I developed the confidence to do all these things.

I am working as a Medical Director of my medical college. So I am actively involved in curriculum development as well as mentoring of the students and also making faculty development programs. So it is also helping me to develop managerial skills.

**Q: If we dig a level deeper, what was it about these experiences that made them a positive learning experience?**

Level of autonomy was very helpful in developing leadership skills. When you are working independently and you are dealing with your colleagues and when you are teaching and helping your colleagues, it really helps you a lot.

**Developmental assignments shaping experience-driven acquisition of leadership competencies in clinicians.**

**Interview from R12**

**Q: What do you understand by leadership?**

Leadership is basically having a vision and making others follow that vision. Clinical leadership is when you take the responsibility of your clinical work and you provide protection to your juniors, you take responsibility of your juniors and you train them with something in your mind that you give them such job assignments that they can handle and them you will be responsible for it. This is a difficult task in that you don’t directly work with them, still you know which person can perform the tasks of what level and then you have to take the responsibility of his work. So basically taking responsibility and training your juniors is what clinical leadership encompasses.

**Q: What, in your opinion, are the leadership qualities in you that you rely on at your workplace?**

I take the responsibility of my work. Whenever my juniors are in a problem, I take over the task and try to make out a decision. Wherever the problem arises, either in diagnosis or in surgery, I take over the work and own the responsibility. I train my juniors in various ways: clinical, surgical, ethical. I think that I am honest and I can trust my juniors and I am able to identify the strengths and weaknesses of my trainees, for example, what are their qualities that I can trust and assign them jobs accordingly.

**Q: Literature says that 70% of leadership is developed by developmental assignments (on-the-job tasks). What developmental assignments can you think of, that you would credit, from which you acquired the aforementioned leadership qualities?**

I learned a lot of leadership from my coach and mentor. I used to observe her during my house job and training and she also used to guise us from the beginning that be a leader from the beginning. It is not necessary that you become a professor and only then you become a leader. You can become a leader even in your house job. Another thing that she used to say was that there is no job which cannot be done. So never say no to any work and try to find out ways to do that. Whenever she would give me any tasks, I used to find out ways to do what things I thought that I could do. She used to give me very difficult tasks like beyond my level. My professor also used to give me tasks beyond my level during house job. I used to work a lot and I used to try to find out ways to do them like asking the seniors, searching about the knowledge component of the task and then I used to complete it and give it to them. Then they used to give me feedback and after that, they would ask me to do the task again and again. When it was perfect only then they would accept it. I remember during my training, there used to be one junior resident and one senior resident on duty. I used to take all the responsibility of the labor room and clinical room as a junior resident We used to do 24 hour duties every alternate day. So staying at the workplace a lot helped me in developing leadership. I used to go home for only ten hours. I knew all the patients, for example, when they came and then I would follow them throughout till the end when they would be discharged. This is contrary to the practice today where residents just do their part of the duty and do not worry about the outcome of the patient. Every patient is a developmental task. When I was a junior resident, I used to ask my senior resident to stay in her office and I will tell you about all the patients. I will examine them all myself, evaluate them, devise a management plan and manage them and will inform you. I think I learned a lot because of doing everything myself. I was never too eager about surgery. I was more concerned with diagnosis and clinical work but alhamdulillah I also gained surgical competencies because of a lot of practice. Even now when I have become a senior, I learn with every C-section that I do and try to improve my own self by evaluating every surgery and every task and try to self-improve. I was also given research-oriented tasks during residency. My seniors used to just tell me what the task was and I had to find out my own ways and means to do that. That has helped me a lot as now I do not consider anything impossible. During residency, I used to teach my juniors and house officers. I used to teach my juniors about the tests that we would get. We were involved in managerial tasks in the fourth year of residency. We used to have greater responsibility even than the senior registrars. The fourth year trainees were called Gynae 1. So instead of the senior registrars, Gynae 1 was more accountable to the seniors. All the managerial tasks, for example, of wards, labs, communication with other departments, coordination was done by us. I think experience gives you more leadership qualities but the first inspiration you get, is from your mentors even if it is just a sentence or small tasks. If they don’t give you tasks more your level, even you will not be able to explore your strengths and qualities.

**Q: If we dig a level deeper, what was it about these experiences that made them a positive learning experience?**

Q: High levels of responsibility: Do you think that high level of responsibility in a task can help in developing leadership skills in the person performing the task? Also, how does this occur and can you relate to it in the clinical scenario?

I was always given the tasks which were of a higher level. This was probably one of the reasons I developed my leadership skills. Every person is not given higher level tasks. When I assess my trainees, as in, who to give higher level tasks, we see the characteristics of the trainee, do they take responsibility, do they fulfil their tasks, do they work hard enough that they will be able to complete it properly. Personal characteristics also matter. All the trainees are given tasks according to their level, for example, if a second year trainee is asked to do a C-section, then this task is according to his/her level as all the second year trainees are doing C-sections. Even from an academic point of view, if you give a trainee a task such as conducting the final year exam, then we assess them based on their characteristics such a s honesty, time management, whether they can perform the task without making a hustle, whether they can maintain secrecy. Then they are given the task if they manage it well, then they are given a higher level task and so on. The issue of fairness did not arise because my fellow residents were always very happy that they were not given extra work. Surgical competency is an entirely different thing where personal capabilities and characteristics matter a lot. Some people learn only by observing. They are inborn surgeons and you do not have to take them by the hand or teach them step by step. We were trained to do C-sections and hysterectomies under supervision. The higher level surgeries were learned by observation.

Q: Creating change: How do you think a task involving creating change of any sort will affect leadership development in the person performing the task?

During training, I used to think about changing the surgical techniques to improve them, for example, previously we did not use to close the peritoneum after a C-section. I proposed in my department that a lot of problems arise due to not closing the peritoneum. Then I went into the details of the research regarding this and soon there were studies that proved that closing the peritoneum was better. Our mentors also recognized this and implemented this change. Such small changes were a apart of my training but changes of a bigger magnitude were brought on at senior registrar and above level. If you do not bring change, you will not be able to bring improvement in any unit. You have to bring a a change for an improvement.

Q: Unfamiliar responsibilities: Do you think that a task involving unfamiliar responsibilities can help in leadership development? Can you relate to this from your training?

All the tasks were unfamiliar and I used to research myself, ask seniors, find out who had done that task before and make my own way. Today I can do a lot of tasks because of those exercises that I went through during my training.

Q: Working across boundaries: How do you think working across boundaries helps in leadership development in clinical trainees?

My communication has always been very good with all the departments, nursing staff and paramedical staff. There used to be big conflicts for surgeries but I was never involved in any of them as I would always get the operating table for my surgery due to my communication skills. Now I also collaborate with higher level staff.

Q: Managing diversity: How do you think a task involving managing diversity can affect leadership development in the clinical trainees performing the task?

I know how to handle different types of people in different ways. I learned this from a professor who came to our unit for a short period of time. I observed her to learn how to deal with different types of patients, aggressive patients, and different types of residents. You learn this at the workplace and also by your mentor. You learn from the response you get from the people. In the beginning, you make mistakes but then you go back and evaluate and improve yourself slowly. This takes a lot of time.

Q: What do you think should the level of autonomy while assigning a task?

I was given a lot of autonomy during my first year of residency, so I am a firm believer that autonomy should be given to the trainees. But autonomy has to accompanied by audit. You should be supervised according to the level of the trainee. Slowly the trainees should be given the confidence that they can do the job independently.

**Q: As a clinical leader, you assign tasks to you subordinates/juniors. Do you consider incorporating certain elements/characteristics in those tasks in order to help the assignees grow as leaders?**

When we discuss morning meeting and are discussing different patient cases, we ask the senior residents to take the responsibility of junior residents, supervise and train them and teach them how to manage different managerial task and different problems. We also teach them ethics, team work and inter-departmental communication. When we do rounds, we tell them how to deal with patients.

Q: What, in your view, is the effect of Briefing before assigning a task & Debriefing after the completion of the task on leadership development of the trainee performing the task?

I never used to get any briefing before a task. I learned everything on my own. Briefing before a task is important as it makes the task easier for the trainee. However, I think that it is not essential for leadership development. However, not every person can do the job without a briefing. This is what I have observed here that you need to give a lot of briefing before a task. Nobody here can do the job well without a detailed briefing. I feel that my mentors used to give me tasks without a detailed briefing. Some briefing should however be given. After the task, I used to get a lot of feedback and that was very important in leadership development. Now I have trainees who are working on their synopsis or dissertations and I say to them that I will not tell you the research topic myself. You have to find your area of interest yourself and then I will guide you how to look for a research problem and find your topic. I do not provide them answers to the questions directly myself, rather I ask them to go and search for the answers. They come to them many times with their topics, each time we discuss and then we modify it. Similarly in synopsis, I do not give them answers directly. I tell them where to search it from. I guide them, they do it themselves and bring it back to me and then I give them feedback. If you start spoon feeding, then the trainees do not use their own creativity at all.

**Developmental assignments shaping experience-driven acquisition of leadership competencies in clinicians.**

**Interview from R13**

**Q: What do you understand by leadership?**

I think specifically clinical leadership involves clinical care where you have to make on the spot decisions and because you are dealing with life and death situations and the stakes are very high. So I think leadership at that point is to make the right decisions at the right point. That is the most frequent task that we have to accomplish as a leader.

**Q: What, in your opinion, are the leadership qualities in you that you rely on at your workplace?**

For myself, I would say my commitment to my work and I think being a role model using that commitment and ensuring that others also see that commitment for others to follow.

**Q: Literature says that 70% of leadership is developed by developmental assignments (on-the-job tasks). What developmental assignments can you think of, that you would credit, from which you acquired the aforementioned leadership qualities?**

As such I don’t recall any structural things. I think it was more on the job things and observing others and learning what is good in others and adopting that. During postgraduate training, you are actually given responsibility gradually over the time, I would consider it more towards something structured. But formally saying that doing it or having specific tasks, I don’t as such recall. I think assignments like, doing something for the seniors, for example, making their presentations or organizing something for them, participating or co-facilitating helped in leadership development. In the postgraduate training, gradually as you are acquiring more competencies, you are getting more leverage to do things and then get back to your supervisors and getting it checked and getting their feedback on clinical tasks. I also think taking academic classes of the paramedics or solving problems of the staff in the wards or dealing with the junior trainees or house officers as a senior resident helped in leadership development.

**Q: If we dig a level deeper, what was it about these experiences that made them a positive learning experience?**

Once you have done a task, you start to have a command over things and you get the confidence that I have done this task so I was able to manage it, the feeling of fulfillment helps in leadership development. Secondly, getting a positive feedback from your seniors, that would have helped in leadership development that yes, you have done this well. Sometimes if you have not done something right, then getting feedback is also important.

Q: What do you think should the level of autonomy while assigning a task?

I would consider myself a very conscientious and responsible person any way so any task that was given to me, I always took it very seriously. Gradually, you are given increased level of responsibility as you go along your training. So I think increasing responsibility has helped me a lot.

**Q: Certain characteristics of tasks that help in leadership development have been identified in the corporate sector. Can you please elaborate on and relate to them with respect to clinical tasks?**

Q: High levels of responsibility: Do you think that high level of responsibility in a task can help in developing leadership skills in the person performing the task? Also, how does this occur and can you relate to it in the clinical scenario?

Level of autonomy will depend on their stage of training. If they are a very junior trainee, sometimes you don’t give them the whole autonomy. You ask them to come and give you a feedback and then go along. As their training progresses and they are more experienced in their training and they have more exposure, then their level of autonomy definitely increases. So in fourth year, I would give a whole case to a trainee and let them be responsible for that.

Q: If you feel that a first year resident has the caliber of performing a second or a third year resident’s tasks and you feel that doing so will enhance leadership development in that trainee, will you assign a higher level task to him/her?

I would give the trainee a higher level task, but keeping patient safety in mind. There are always people who perform better than the others and I give them higher levels of responsibility.

Q: Creating change: How do you think a task involving creating change of any sort will affect leadership development in the person performing the task?

I would not say that I have been part of a major change. I think I have experienced minor changes only like how things are organized and giving feedback and expressing your opinions about things. When I joined this institute, here gradually things have changed in the sense that how the training of the trainees has been organized, that has evolved over the years, how the undergraduates are exposed to psychiatry, that has also changed to a certain extent. There has not been any major change because of a lot of restrictions from the administration, but academically more so.

Q: Unfamiliar responsibilities: Do you think that a task involving unfamiliar responsibilities can help in leadership development? Can you relate to this from your training?

Unfamiliar responsibilities are a part of our everyday job tasks. Definitely when you start things from scratch and you achieve them in the end, you feel more confident and you feel able of yourself.

Q: Working across boundaries: How do you think working across boundaries helps in leadership development in clinical trainees?

I think collaborating with different departments on different tasks is a part of our job. As a clinician, you tend to do that with your patients and academically as well. So there are a lot of things going on with medical people. An example would be regarding the undergraduate teaching, how they are exposed to behavioral sciences and collaborating with different departments for incorporating behavioral sciences in their academics.

Q: Managing diversity: How do you think a task involving managing diversity can affect leadership development in the clinical trainees performing the task?

We deal with diverse groups. I would say that we have been able to or we are very frequently working with diverse groups in the sense that they are all together.

**Q: As a clinical leader, you assign tasks to you subordinates/juniors. Do you consider incorporating certain elements/characteristics in those tasks in order to help the assignees grow as leaders?**

I thinks setting timelines for those tasks, asking for feedback where they think they are stuck, ensuring and being there for them whenever they need help to achieve a task or if they are stuck with some task, facilitating them to get it done which might involve collaborating with other departments. I think being accountable is a leadership quality and as a leader, you should be internally motivated to do things but accountability has to be there for the task to be accomplished in the right way and not the things happening which might be not very fair or just at times.

Q: What, in your view, is the effect of Briefing before assigning a task & Debriefing after the completion of the task on leadership development of the trainee performing the task?

I think briefing and debriefing are very important. Briefing would be give them an outline and debriefing would give them a feedback that how easy or how difficult it was and what could have been done to change that or to make it easier.

**Developmental assignments shaping experience-driven acquisition of leadership competencies in clinicians.**

**Interview from R14**

**Q: What do you understand by leadership?**

Leadership means that you have to guide others but you also have to make a team so that you can delegate responsibilities to others. The whole team has to work together to achieve the goal. So leader is one person who will make the team to achieve that goal.

**Q: What, in your opinion, are the leadership qualities in you that you rely on at your workplace?**

First of all, hard work, diligence and then communication skills are the leadership qualities that I think I have in me and I use them at my workplace.

**Q: Literature says that 70% of leadership is developed by developmental assignments (on-the-job tasks). What developmental assignments can you think of, that you would credit, from which you acquired the aforementioned leadership qualities?**

I think I developed leadership skills during my FCPS training. My supervisor used to send me for final year classes. That really helped build confidence in me. Then he used to entrust us with surgical procedures. That also you know gave us a confidence boost.

**Q: If we dig a level deeper, what was it about these experiences that made them a positive learning experience?**

My supervisor’s trust in my abilities that I can do these tasks on my own pushed me to achieve difficult tasks.

One more thing is that I had to collaborate with inter-disciplinary teams that also developed leadership skills in me, like working with staff and students from different backgrounds.

**Q: As a clinical leader, you assign tasks to you subordinates/juniors. Do you consider incorporating certain elements/characteristics in those tasks in order to help the assignees grow as leaders?**

In morning meetings and rounds, I give patients’ responsibility to the senior residents and also the responsibility of junior residents to supervise and train them. We also teach them ethics, team work and inter-departmental communication. I also give them a free hand while giving them a task. I give them a goal set by collaborating with them.
